# Exosomal transfer of long non-coding RNA SBF2-AS1 enhances chemoresistance to temozolomide in glioblastoma

**DOI:** 10.1186/s13046-019-1139-6

**Published:** 2019-04-16

**Authors:** Zhuoran Zhang, Jianxing Yin, Chenfei Lu, Yutian Wei, Ailiang Zeng, Yongping You

**Affiliations:** 1 Department of Neurosurgery, The First Affiliated Hospital of Nanjing Medical University, Nanjing, 210029 China; 2 Jiangsu Key Lab of Cancer Biomarkers, Prevention and Treatment, Jiangsu Collaborative Innovation Center For Cancer Personalized Medicine, Nanjing Medical University, Nanjing, 211166 China

**Keywords:** Exosomes, LncRNA-SBF2-AS1, Temozolomide-resistance, Glioblastoma

## Abstract

**Background:**

Acquired drug resistance is a constraining factor in clinical treatment of glioblastoma (GBM). However, the mechanisms of chemoresponsive tumors acquire therapeutic resistance remain poorly understood. Here, we aim to investigate whether temozolomide (TMZ) resistance of chemoresponsive GBM was enhanced by long non-coding RNA SBF2 antisense RNA 1 (lncRNA SBF2-AS1) enriched exosomes.

**Method:**

LncSBF2-AS1 level in TMZ-resistance or TMZ-sensitive GBM tissues and cells were analyzed by qRT-PCR and FISH assays. A series of in vitro assay and xenograft tumor models were performed to observe the effect of lncSBF2-AS1 on TMZ-resistance in GBM. CHIP assay were used to investigate the correlation of SBF2-AS1 and transcription factor zinc finger E-box binding homeobox 1 (ZEB1). Dual-luciferase reporter, RNA immunoprecipitation (RIP), immunofluorescence and western blotting were performed to verify the relation between lncSBF2-AS1, miR-151a-3p and XRCC4. Comet assay and immunoblotting were performed to expound the effect of lncSBF2-AS1 on DNA double-stand break (DSB) repair. A series of in vitro assay and intracranial xenografts tumor model were used to determined the function of exosomal lncSBF2-AS1.

**Result:**

It was found that SBF2-AS1 was upregulated in TMZ-resistant GBM cells and tissues, and overexpression of SBF2-AS1 led to the promotion of TMZ resistance, whereas its inhibition sensitized resistant GBM cells to TMZ. Transcription factor ZEB1 was found to directly bind to the SBF2-AS1 promoter region to regulate SBF2-AS1 level and affected TMZ resistance in GBM cells. SBF2-AS1 functions as a ceRNA for miR-151a-3p, leading to the disinhibition of its endogenous target, X-ray repair cross complementing 4 (XRCC4), which enhances DSB repair in GBM cells. Exosomes selected from temozolomide-resistant GBM cells had high levels of SBF2-AS1 and spread TMZ resistance to chemoresponsive GBM cells. Clinically, high levels of lncSBF2-AS1 in serum exosomes were associated with poor response to TMZ treatment in GBM patients.

**Conclusion:**

We can conclude that GBM cells remodel the tumor microenvironment to promote tumor chemotherapy-resistance by secreting the oncogenic lncSBF2-AS1-enriched exosomes. Thus, exosomal lncSBF2-AS1 in human serum may serve as a possible diagnostic marker for therapy-refractory GBM.

**Electronic supplementary material:**

The online version of this article (10.1186/s13046-019-1139-6) contains supplementary material, which is available to authorized users.

## Introduction

Malignant glioma is the most common primary malignant tumor in the brain as well as being the most lethal in adults. The alkylating drug temozolomide (TMZ) is the international standard chemotherapy for the treatment of glioblastoma multiforme (GBM) [1]. However, resistance to TMZ and subsequent tumor recurrence has become difficult and critical problems in the clinical treatment of GBM. Therefore, exploring the mechanisms underlying acquired TMZ resistance could offer promising new molecular targets for GBM treatment.

Exosomes are the most clearly defined vesicles known so far, with diameters ranging from 40 nm to 100 nm, and can be released from a variety of cell types, such as T-lymphocytes, neurons, and GBM cells [2]. Recently, ever more attention has been paid to their potential diagnostic and therapeutic values, especially in tumors [3–5]. In general, most studies on tumor drug resistance have focused on tumor cells themselves. Genetic changes enhancing metabolic activities that inactivate drugs, oncogene activation, tumor suppressor gene inactivation, tumor heterogeneity development, and defects in apoptosis mechanisms have been found to improve drug resistance. Exosomes secreted by tumor cells serve as a key mediator of tumor communication between tumor cells or with other cells in the microenvironment. Hence, it has emerged as a new model for research into tumor resistance and become a new hotspot in the study of drug resistance in tumor biology.

Long non-coding RNA (lncRNA) is a non-coding RNA longer than 200 nucleotides in length. Studies have shown that long non-coding RNAs are abnormally expressed in cancer cells and are related to cancer progression [6] including apoptosis [7], metabolism [8], proliferation and metastasis [9]. Salmena and colleagues firstly propounded lncRNAs can act as a competitive endogenous RNA (ceRNA), which posited that lncRNAs derepress the level of miRNA-targeted mRNAs via sponge miRNAs [10, 11]. Based on this hypothesis, the “lncRNA-miRNA-mRNA” axes have been explored in multiple cancers including GBM [12]; however, the effect of ceRNA network in GBM progression especially on drug resistance is still unclear.

In this study, we sought to investigate the effects of exosome-transmitted lncRNA SBF2-AS1 on the molecular mechanism underlying TMZ resistance in GBM. The finding of this study may serve to develop a potential therapy for TMZ-resistant GBM.

## Materials and methods

### Clinical specimens

See Additional file 1: Supplementary materials and methods for details on patient samples information. The study was approved by the Ethics Committee of Nanjing Medical University and written informed consent was obtained from all patients.

### Cell culture

Five human GBM cell lines (U87, LN229, A172, T98, U251) and human embryonic kidney (HEK) 293 T cells were purchased from the Chinese Academy of Sciences Cell Bank (Shanghai, China), N3 primary culture cell were donated from Tian Tan Hospital and four drug-related cell lines (Pri GBM, N3S, Rec GBM, N3T3rd) were used from our previous study [13]. All GBM cells were maintained in high glucose DMEM medium supplemented with antibiotics (100 units/mL penicillin and 100 mg/mL streptomycin) and 10% fetal bovine serum (FBS). Normal human astrocytes (NHAs) were acquired from Sciencell Research Laboratories (Carlsbad, CA, USA) and cultured in astrocyte medium (Carlsbad, CA, USA). All cells were grown at 37 °C with 5% CO_2_.

### Exosome isolation

Exosomes were extracted from GBM cell culture through standard centrifugation steps as previously described [14]. See Additional file 1: Supplementary materials and methods for details on exosome isolation.

### RNA extraction and quantitative reverse transcription (qRT) -PCR assays

See Additional file 1: Supplementary materials and methods.

### Western blot analysis

Western blot analysis was done as described previously [15]. Antibodies against caspase-3, ZEB1 (Cell Signaling Technology, USA); CD63, CD81, GM130, XRCC4 and γ-H2AX (Abcam, USA); and β-actin, GAPDH and tubulin (Santa Cruz, USA) were used for Western blot analysis.

### Plasmid construction, transfection, and stable cell establishment

See Additional file 1: Supplementary materials and methods for details on transfection and stable cell establishment.

### Fluorescence in situ hybridization (FISH) and immunohistochemistry (IHC) analysis

Fish analysis was performed on human tissue and GBM cells using the method described previously [16]. IHC was performed on mice xenogeneic tumor tissue and human tissue as described previously [16].

### Flow cytometric analysis

The flow cytometric assay was performed as described previously [13]. The apoptotic cells were stained with Annexin V-FITC/PI and assessed by fluorescence activated cell sorting (FACS).

### Colony formation assay

The colony formation assay was performed following the method described in our earlier study [17]. Cells were independently seeded onto 6-well plates followed by treatment with TMZ (200 μM, 24 h). Then the culture media were replaced by TMZ-excluded media. After 10–15 days of growth, cells were fixed with 4% paraformaldehyde and stained with crystal violet.

### Immunofluorescent staining

See Additional file 1: Supplementary materials and methods for details on immunofluorescent staining.

### TUNEL assay

GBM cells were fixed in 4% paraformaldehyde for 15 min and stained with In Situ Cell Death Detection Kit, POD (Roche, Switzerland) according to the manufacturer’s instructions. Tunel-positive cells were observed with a Nikon ECLIPSE E800 fluorescence microscope.

#### RNA immunoprecipitation (RIP)

RIP™ RNA-Binding Protein Immunoprecipitation Kit (Millipore, Billerica, MA, USA) was used for RIP. GBM cell lysates were prepared and incubated with RIP buffer containing magnetic beads conjugated with human anti-Argonaute2 (anti-Ago2) antibody (Millipore, Billerica, MA, USA) while normal mouse IgG (Millipore, Billerica, MA, USA) functioned as a negative control. Immunoprecipitated RNA was tested by qRT-PCR analysis to verify the presence of lncSBF2-AS1 using specific primers.

### Luciferase assay

See Additional file 1: Supplementary materials and methods for details on luciferase assay.

### Chromatin immunoprecipitation (ChIP)

Chip assay was performed using Simple Chip Enzymatic Chromatin IP kit (Cell Signaling Technology, USA) according to the manufacturer’s instructions. Briefly, the chromatin of the GBM cells were treated with 3 μg anti-ZEB1 (Abcam). Immunoprecipitated DNA was subjected to RT-PCR using specific primers.

### Alkaline comet assay

Comet assay was performed as described preciously [13]. Briefly, GBM cells that received TMZ treatment (200 μM) were analyzed by DNA Damage Detection Kit (KeyGEN BioTECH, China). Agarose-embedded cells were lysed and examined by agarose gel electrophoresis.

### Subcutaneous and orthotopic xenograft studies

See Additional file 1: Supplementary materials and methods for details on Subcutaneous and orthotopic xenograft studies. All animal experiments were approved by the Institutional Committee for Animal Research of Nanjing Medical University and conducted in conformation with the Animal Welfare Act.

### Statistical analysis

All statistical analyses were done using GraphPad Prism 5 Software (La Jolla, CA, USA). All values were shown as mean ± Standard Error of Mean (SEM) of three independent experiments. Student’s t-test or ANOVA was performed to evaluate pairwise comparison or multivariate analysis. All statistical analyses were performed using SPSS statistics 22 software. *P* value < 0.05 was judged to be statistically significant.

## Result

### LncSBF2-AS1 is upregulated in GBM cell lines and tissues after TMZ treatment

Our previous experimental results, have demonstrated that miR-151a-3p promotes TMZ sensitivity in GBM cells [18]. Recent studies have shown that lncRNAs can act as sponges or function as competing endogenous RNA (ceRNA) to regulate miRNA expression [19, 20]. Thus, we considered whether miR-151a-3p is regulated by lncRNA. We used the bioinformatic website (DIANA TOOLS, http://diana.imis.athena-innovation.gr/) to find potential lncRNAs that could regulate miR-151a-3p. We chose the top 10 lncRNAs predicted in the website and verified their expression levels in N3 GBM cells by qRT-PCR (Additional file 2 Figure S1A). The result showed that five lncRNAs (RP11-111F5.4, PSMD-AS1, RP11-967 K21.1, SBF2-AS1, ARHGEF26-AS1) had higher expression in N3 GBM cells. We then used the dual luciferase reporter assays to verify the prediction analysis. HEK293T cells were transfected with luciferase plasmid containing the sequence of miR-151a-3p together with plasmids carrying the lncRNAs or control sequence. We found that lncSBF2-AS1 had the strongest ability to suppress miR-151a-3p-driven luciferase activity (Additional file 2 Figure S1B). Meanwhile, Knockdown of SBF2-AS1 in N3 GBM cells had the highest caspase-3 activity (Additional file 2 Figure S1C). Then we examined the levels of SBF2-AS1 in normal human astrocytes (NHA) and five GBM cell lines (U87, LN229, A172, T98, U251) using qRT-PCR. All GBM cells were found to highly express SBF2-AS1 in comparison with NHAs (Additional file 2 Figure S1D). Next, we analyzed lncSBF2-AS1 in TCGA database of GBM and found that lncSBF2-AS1 level was higher in GBM than low-grade gliomas (LGG), and high SBF2-AS1 expression correlated with poor survival in GBM patients (Additional file 2 Figure S1E and F). Thus, we considered lncSBF2-AS1 as the upstream of miR-151a-3p and lncSBF2-AS1 was chosen as a candidate for further investigation.

**Fig. 1:**
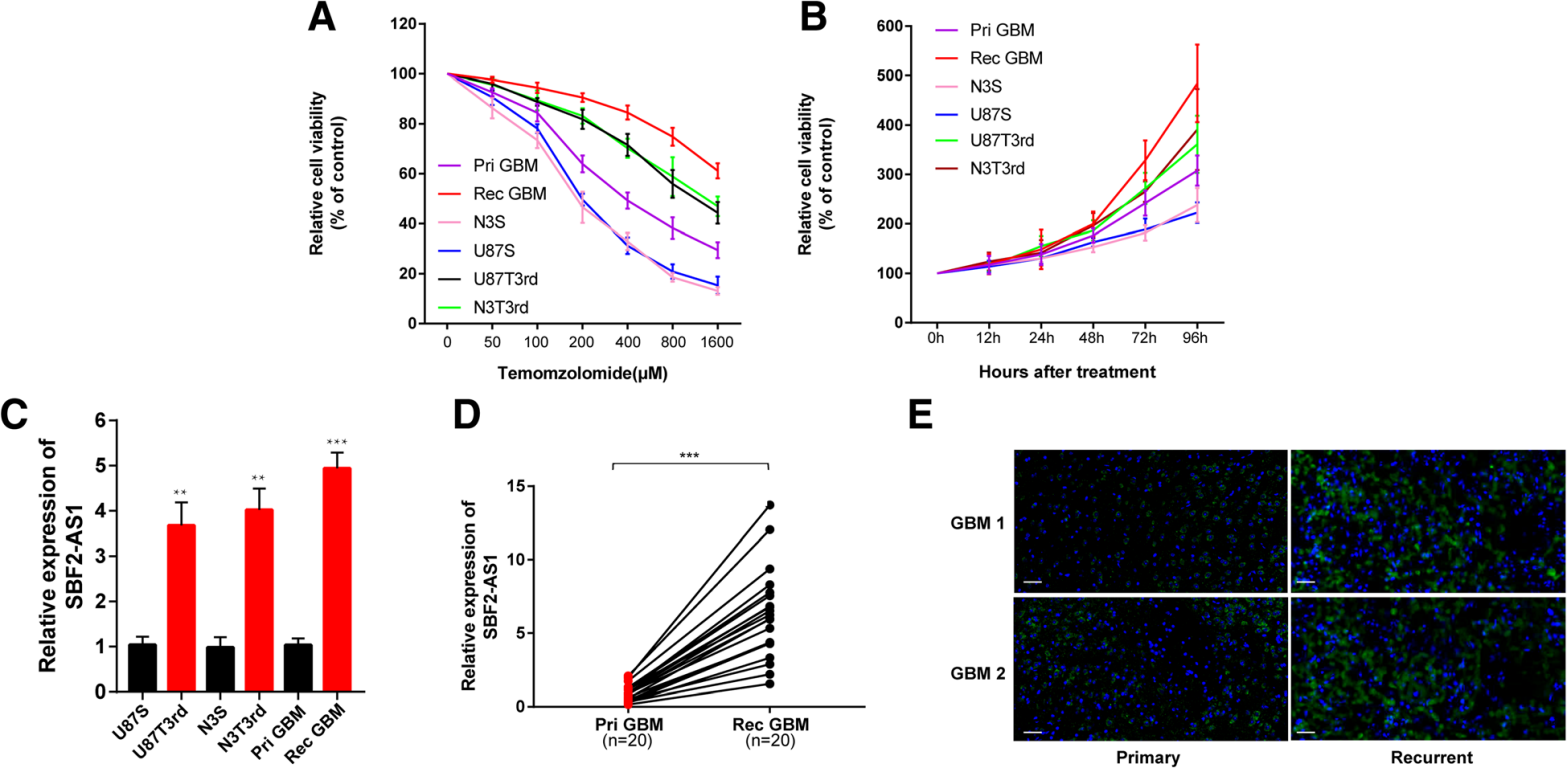
LncSBF2-AS1 is upregulated in GBM cell lines and tissues after TMZ treatment. **a** Cell proliferation of 6 GBM cells was evaluated in response to TMZ at different doses. CCk8 assay was performed after 48 h TMZ treatment. Pri GBM (IC50 = 423.33 μM), N3S (IC50 = 187.73 μM), U87S (IC50 = 197.64 μM), N3T3rd (IC50 = 1321.92 μM), U87T3rd (1143.09 μM). Data are presented as mean ± SEM. **b** Cell proliferation was eveluated in 6 GBM cells in 200 μM TMZ treatments using CCK8 assay. Data are presented as mean ± SEM. **c** Expression of LncSBF2-AS1 analyzed by qRT-PCR in TMZ resistant cells and TMZ sensitive cells. The data represents the mean ± SEM from three independent experiments. **P < 0.01, ***P < 0.001 (**d)** Analysis of LncSBF2-AS1 expression by qRT-PCR in 20 primary GBM tissues and 20 recurrent GBM tissues. Data represents means of three independent experiments ± SEM. ***P < 0.001 **(e)** FISH analysis of LncSBF2-AS1 expression in recurrent tumor tissues compared to primary tumor tissues. Scale bar, 50 μm

To confirmed the effect of SBF2-AS1 on chemoresistance, we first analyzed TMZ-resistance and TMZ-sensitive GBM cell viability in response to TMZ (Fig. 1a and b). The levels of SBF-AS1 in TMZ-sensitive cell lines and TMZ-resistant cell lines, analyzed by qRT-PCR, showed that TMZ resistant cells had higher SBF2-AS1 levels (Fig. 1c). Then we measured SBF2-AS1 levels in 20 primary GBMs and 20 recurrent GBMs by qRT-PCR (Fig. 1d). Fluorescence in situ hybridization (FISH) assays employing SBF2-AS1 probe showed that recurrent GBM tissues displayed higher level of SBF2-AS1, whereas primary GBM tissues exhibited lower SBF2-AS1 signal (Fig. 1e). Together, these results suggest that lncSBF2-AS1 is upregulated in TMZ-resistant GBM cell lines and tissues, pointing towards a possible correlation between SBF2-AS1 and TMZ resistance.

### LncSBF2-AS1 overexpression confers TMZ resistance

**Fig. 2:**
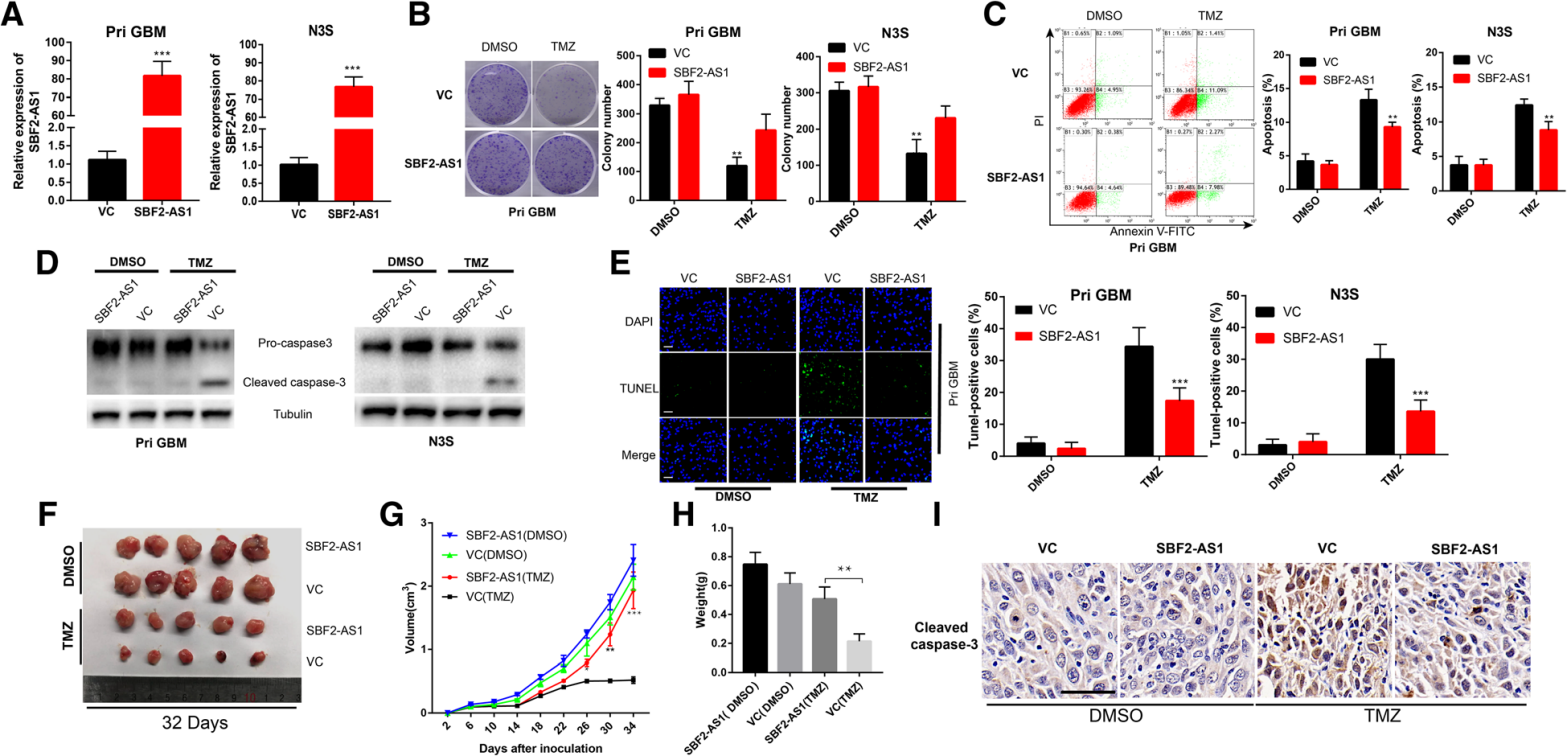
LncSBF2-AS1 overexpression confers TMZ resistance. **a** LncSBF2-AS1 transfected Pri GBM and N3S cells and elevated SBF2-AS1 expression. The data represents the mean ± SEM from three independent experiments. ***P < 0.001. **b** Colony formation ability of SBF2-AS1 overexpressing TMZ-sensitive cells or vector control cells in the absence or presence of TMZ. Data represents mean of three independent experiments ± SEM. ***P < 0.001. **c** Flow cytometry was used to measure the apoptosis of TMZ-sensitive SBF2-AS1 overexpression cells or vector control cells after exposure to 200 μM TMZ for 48 h. Data represents means of three independent experiments ± SEM. ***P < 0.001. **d** Western blot analysis of cleaved caspase-3 expression of TMZ-sensitive cells overexpressing SBF2-AS1 or vector control cells in the absence or presence of TMZ. Tubulin was used as the loading control. **e** TUNEL analysis of TMZ-sensitive cells overexpressing SBF2-AS1 or vector control cells in the absence or presence of TMZ for 48 h. Data represents means of three independent experiments ± SEM. ***P < 0.001. Scale bar, 50 μm. **f** Representative images of Pri GBM cells overexpressing SBF2-AS1 or vector control cells-derived subcutaneous tumors on 42nd day in the absence or presence of TMZ. **g** Growth curve of tumor xenografts originated from SBF2-AS1-overexpressing Pri GBM cells or vector control cells in the absence or presence of TMZ. Data represents means of three independent experiments ± SEM.*P < 0.05, **P < 0.01, ***P < 0.001. **h** Weight of tumor xenografts originated from SBF2-AS1-overexpressing Pri GBM cells or vector control cells in the absence or presence of TMZ. Data represents means of three independent experiments ± SEM. **P < 0.01. **i** IHC analysis of cleaved caspase-3 in SBF2-AS1-overexpressing Pri GBM cells or vector control cells-derived subcutaneous tumors in the absence or presence of TMZ. Scale bar, 50 μm

To determine whether lncSBF2-AS1 is associated with TMZ resistance, we overexpressed SBF2-AS1 in N3S and Pri GBM cells (TMZ sensitive cell line, Fig. 1a and b and Fig. 2a). We found that Pri GBM and N3T3rd cells transfected with vector control (VC) showed a significant decrease in colony-forming ability after TMZ treatment, whereas overexpression of SBF2-AS1 in Pri GBM and N3S cells rescued the colony-forming capacity of TMZ (Fig. 2b). We also tested apoptotic rate of TMZ-sensitive cells overexpressing SBF2-AS1 or VC cells in response to TMZ. As shown in Fig. 2c, d and e, a marked decrease in number of apoptotic cells with a co-dependent reduction in apoptosis related protein, cleaved caspase-3 [21], was observed in N3S and Pri GBM cells overexpressing SBF2-AS1 compared with VC cells. These findings indicate that SBF2-AS1 overexpression reverses TMZ-induced apoptosis and growth inhibition in TMZ sensitive GBM cells.

To examine whether lncSBF2-AS1 confers TMZ resistance in vivo, Pri GBM cells expressing SBF2-AS1 or VC was subcutaneously injected into immunocompromised mice. After GBM engraftment was confirmed, we treated mice with TMZ (66 mg/kg/day, 5 days per week for three cycles) or DMSO. We observed that mice injected with Pri GBM-VC had a marked decrease in tumor volume and weight in response to TMZ treatment, whereas xenografts overexpressing SBF2-AS1 had unconspicuous effects on tumor growth when treated with TMZ (Fig. 2f, g and h). IHC staining was performed to verify the expression of cleaved caspase-3. TMZ treated xenografts overexpressing SBF2-AS1 had a significantly decreased level of cleaved caspase-3 compared with control xenografts (Fig. 2i). Hence, our data indicated that overexpression of SBF2-AS1 can lead to the development of acquired TMZ resistance.

### Knockdown of lncSBF2-AS1 sensitizes TMZ-resistant GBM cells to TMZ

**Fig. 3:**
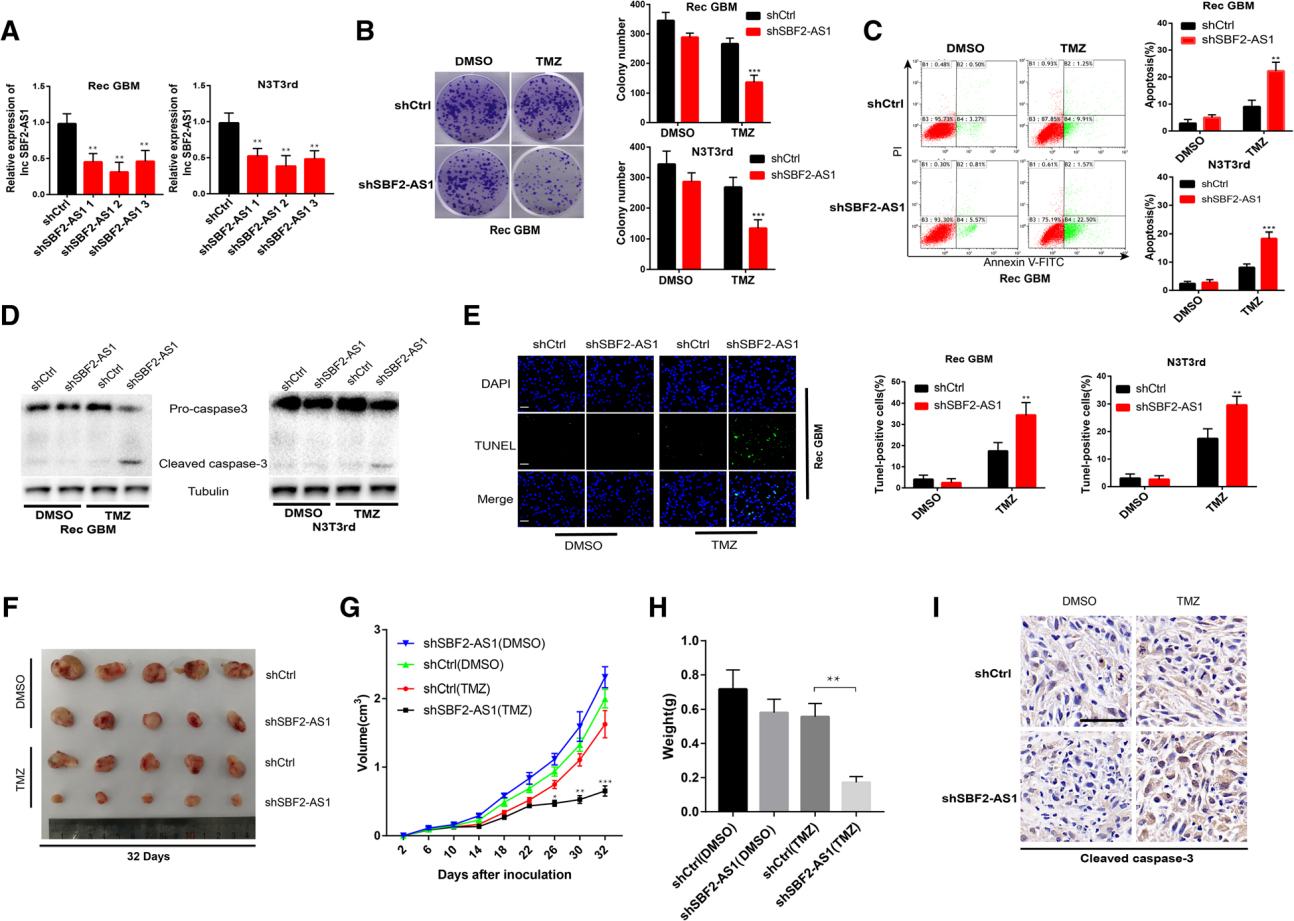
Inhibiting endogenous lncSBF2-AS1 in recurrent GBM cells promotes TMZ-induced cell apoptosis (**a**) SBF2-AS1 knockdown lentivirus transfected Rec GBM and N3T3rd cells showing reduced SBF2-AS1 expression. Data represents means of three independent experiments ± SEM. ***P < 0.001. **b** Colony formation ability of SBF2-AS1 knockdown cells or vector control cells in the absence or presence of TMZ. Data represents means of three independent experiments ± SEM. ***P < 0.001. **c** Flow cytometry was used to measure the apoptosis of SBF2-AS1 knockdown cells or vector control cells after exposure to 200 μM TMZ for 48 h. Data represents means of three independent experiments ± SEM. ***P < 0.001. **d** Western blot test of cleaved caspase-3 expression of SBF2-AS1 knockdown cells or vector control cells in the absence or presence of TMZ. Tubulin was used as the loading control. **e** TUNEL analysis of SBF2-AS1 knockdown cells or vector control cells in the absence or presence of TMZ for 48 h. Data represents means of three independent experiments ± SEM. ***P < 0.001. Scale bar, 50 μm. **f** Representative images of Rec GBM SBF2-AS1-knockdown or shctrl cells-derived subcutaneous tumors on 34th day in the absence or presence of TMZ. **g** Growth curve of tumor xenografts originated from Rec GBM SBF2-AS1-knockdown or shctrl cells in the absence or presence of TMZ. Data represents means of three independent experiments ± SEM. *P < 0.05, ***P < 0.001. **h** Tumor weight of tumor xenografts originated from Rec GBM SBF2-AS1-knockdown or shctrl cells in the absence or presence of TMZ. Data represents means of three independent experiments ± SEM. **P < 0.01. **i** IHC analysis of cleaved caspase-3 in Rec GBM SBF2-AS1-knockdown or shctrl cells-derived subcutaneous tumors in the absence or presence of TMZ. Scale bar, 50 μm

According to the above findings, we determined whether depletion of SBF2-AS1 could cause sensitization to TMZ in GBM cells. We transfected TMZ-refractory recurrent GBM cells and N3T3rd cells (TMZ resistant cell line, Fig. 1a and b) with three separate luciferase-encoding SBF2-AS1 shRNA or control shRNA. The expression of SBF2-AS1 in recurrent GBM cells and N3T3rd cells was analyzed by qRT-PCR (Fig. 3a). According to the knockdown efficiency, we chose shSBF2-AS1–2 for subsequent experiments. SBF2-AS1-knockdown recurrent GBM cells and N3T3rd cells showed a significant decrease in colony-forming ability (Fig. 3b) and the delay in apoptosis was remarkably increased (Fig. 3c, d and e).

Then we investigated the role of SBF2-AS1 deletion on TMZ resistance in vivo. SBF2-AS1-depleted Rec GBM cells or control cells were inoculated subcutaneously into immunocompromised mice. The tumor-bearing mice were given DMSO or TMZ treatment (66 mg/kg/day). As we expected, the growth ability of tumor generated by shSBF2-AS1-expressing GBM cells were significantly lower than those produced by control GBM cells after exposure to TMZ (Fig. 3f, g and h). We also used IHC to measure cleaved caspase-3. SBF2-AS1-knockdown xenografts had an obviously high expression level of cleaved caspase-3 in comparison with control xenografts in response to TMZ (Fig. 3i). These results confirm that inhibition of lncSBF2-AS1 endows TMZ-refractory recurrent GBM cells with chemotherapy sensitivity in vitro and in vivo.

### Transcription factor ZEB1 regulates lncSBF2-AS1 and is associated with TMZ resistance in GBM cells

**Fig. 4:**
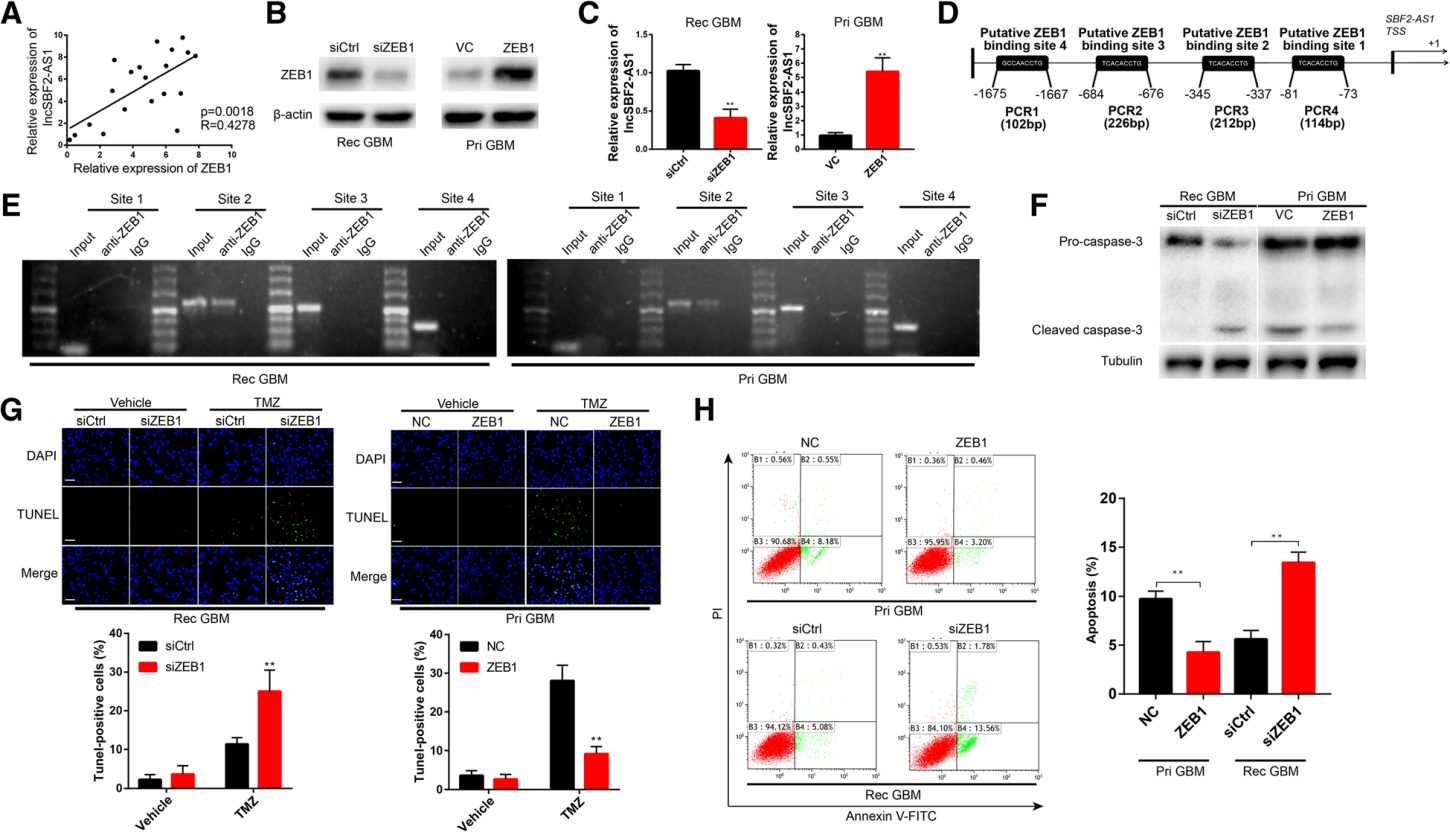
Endogenous ZEB1 regulated SBF2-AS1 at transcriptional level. **a** Each individual ZEB1 and SBF2-AS1 expression was analyzed by linear regression analysis. **b** ZEB1 expression in recurrent ZEB1-knockdown Rec GBM cells and ZEB1-overexpressed Pri GBM cells were analyzed by Western blot assay. β-actin was used as the loading control. **c** SBF2-AS1 expression in Rec GBM cells and Pri GBM cells were analyzed by qRT-PCR. Data represents means of three independent experiments ± SEM. **P < 0.01. **d** Schematic diagram showing the human SBF2-AS1 promoter region. **e** CHIP assay was performed to explore the relative enrichment of ZEB1 on promoter region of lncSBF2-AS1. This figure shows the results. **f** Western blot analysis of cleaved caspase-3 expression of ZEB1-overexpressed Pri GBM cells or ZEB1-knockdown GBM cells in the absence or presence of TMZ. Tubulin was used as the loading control. **g** TUNEL analysis of ZEB1-overexpressed Pri GBM cells or ZEB1-knockdown GBM cells in the absence or presence of TMZ for 48 h. Data represents means of three independent experiments ± SEM. **P < 0.01. Scale bar, 50 μm. **h** Flow cytometry was used to measure the apoptosis of ZEB1-overexpressed Pri GBM cells or ZEB1-knockdown GBM cells after exposure to 200 μM TMZ for 48 h. Data represents means of three independent experiments ± SEM. ***P < 0.001

Several studies have shown that transcription factors can bind to specific regions of promoters to regulate gene expression [22]. Therefore, we explored if transcription factors can regulate SBF2-AS1. Potential transcription factors were predicted using the JASPAR database (http://jaspar.binf.ku.dk/), and luciferase reporter assays were performed to determine the most interactive transcription factor from the top five predictions. Briefly, HEK293T cells were transfected with luciferase plasmid containing the sequence of the SBF2-AS1 promoter region together with plasmids containing the transcription factors or control sequence. The results showed that ZEB1 revealed maximum luciferase activity (Additional file 3 Figure S2A). Then we analyzed ZEB1 expression in TMZ-resistance cells and TMZ-sensitive cells (Additional file 3 Figure S2B). Based on luciferase activities and ZEB1 protein levels in GBM cells, we chose ZEB1 as the object for further study. ZEB1(Zinc finger E-box binding homeobox 1), the key factor of EMT (Epithelial-mesenchymal transition), have been reported as a important molecule for GBM chemoresistance and recurrence [23]. We selected 20 recurrent GBM tissues and correlation analysis between ZEB1 and SBF2-AS1 indicated that the expression level of SBF2-AS1 is correlated with that of ZEB1 (Fig. 4a). To further confirm our hypothesis, we knocked down or overexpressed ZEB1 in Rec GBM or Pri GBM cells, respectively (Fig. 4b), and detected SBF2-AS1 level by qRT-PCR (Fig. 4c). The results showed that SBF2-AS1 level was positively correlated with ZEB1 level. By scanning the promoter region of SBF2-AS1 with the JASPER database, we found four putative ZEB1 binding sites (Fig. 4d). Chromatin immunoprecipitation assay (CHIP) was performed to elucidate the binding of ZEB1 and SBF2-AS1 promoter region. We observed PCR products in the ZEB1 immunoprecipitation in the Site 2 group, which confirmed that ZEB1 bound to SBF2-AS1 promoter at the − 684 to − 676 bp region (Fig. 4e). These data indicated that transcription factor ZEB1 could regulate SBF2-AS1 at transcription level.

In addition, we checked the relationship between ZEB1 and SBF2-AS1 in vitro. The apoptosis of cells was significantly decreased in ZEB1-overexpressed Pri GBM cells as opposed to ZEB1-knockdown Rec GBM cells (Fig. 4f, g and h). These findings indicated that ZEB1 could regulate the level of SBF2-AS1 and was involved in SBF2-AS1-mediated apoptosis of GBM cells.

### LncSBF2-AS1 functions AS a ceRNA and sponges miR-151a-3p in GBM cells

**Fig. 5:**
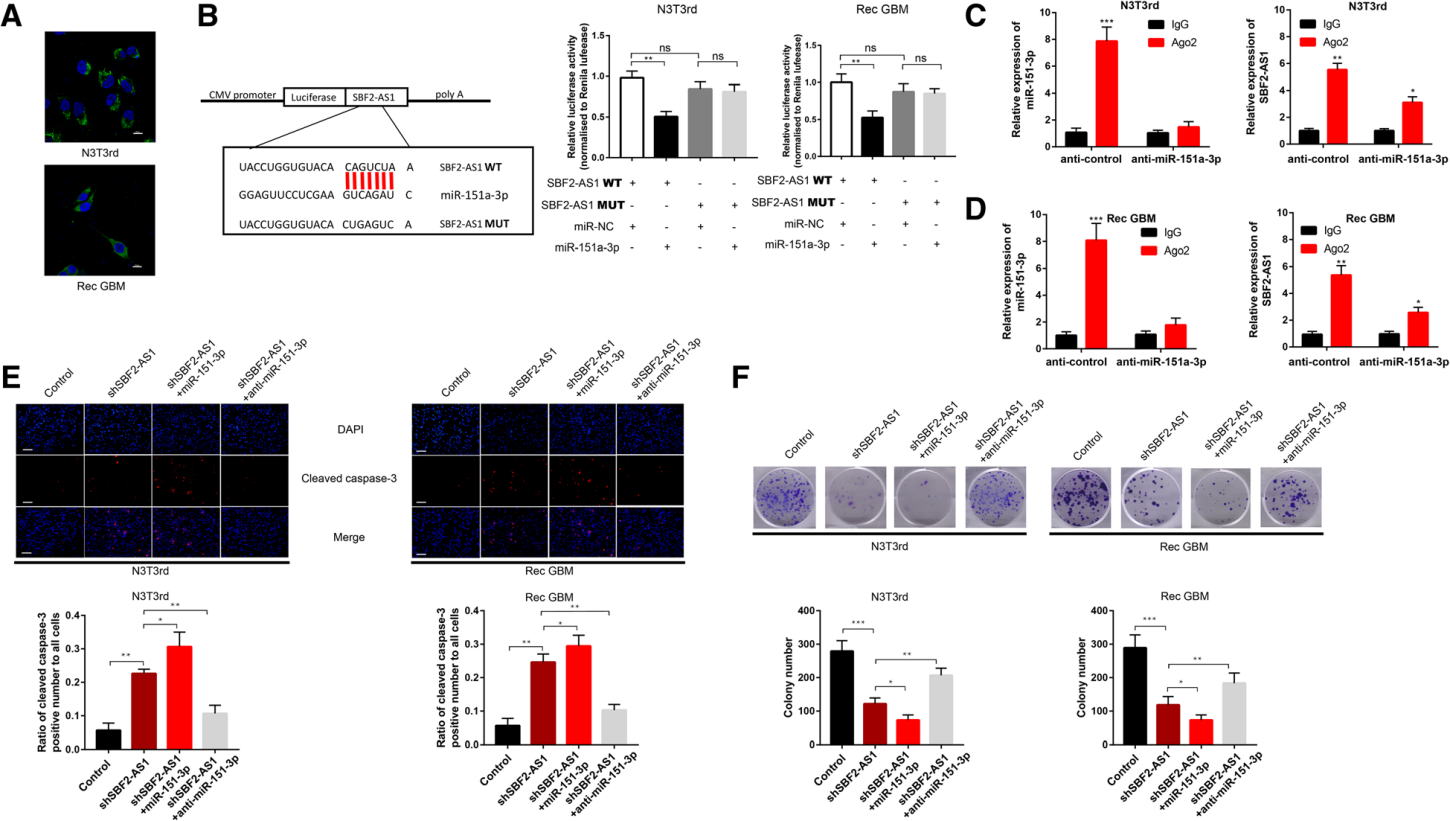
Regulatory relationship between lncSBF2-AS1 and miR-151a-3p (**a**) FISH analysis of the location of lncSBF2-AS1 in the cytoplasm (green) and nucleus (blue) of N3T3rd and Rec GBM cells. **b** Left: Schematic representation of the miR-151a-3p binding sites in lncRNA SBF2-AS1 and the site mutagenesis. Right: The luciferase reporter plasmid carrying wild type (WT) or mutant (MUT) lncSBF2-AS1 was co-transfected into N3T3rd and Rec GBM cells with miR-151a-3p in parallel with an empty vector. Relative luciferase activity in N3T3rd and Rec GBM cells were determined. Data represents means of three independent experiments ± SEM. **P < 0.01. **c** and **d**. RIP assay was established with normal mouse IgG or anti-Ago2 in N3T3rd or Rec GBM cells. Relative expression of SBF2-AS1 and miR-151a-3p were determined by qRT-PCR. Data represents means of three independent experiments ± SEM. **P < 0.01, ***P < 0.001. **e**. Immunofluorescent staining of cleaved caspase-3 in N3T3rd and Rec GBM cells transfected with SBF2-AS1 plasmid, miR-151a-3p mimics + SBF2-AS1 plasmid or miR-151a-3p inhibitor + SBF2-AS1 plasmid after 200 μM TMZ treatment for 48 h. Data represents means of three independent experiments ± SEM. **P < 0.01. Scale bar, 50 μm. **f** Colony formation ability of N3T3rd and Rec GBM cells transfected with SBF2-AS1 plasmid, miR-151a-3p mimics + SBF2-AS1 plasmid or miR-151a-3p inhibitor + SBF2-AS1 plasmid after 200 μM TMZ treatment for 48 h. Data represents means of three independent experiments ± SEM. *P < 0.05, **P < 0.01. Flow cytometry of N3T3rd and Rec GBM cells transfected with SBF2-AS1 plasmid, miR-151a-3p mimics + SBF2-AS1 plasmid or miR-151a-3p inhibitor + SBF2-AS1 plasmid after 200 μM TMZ treatment for 48 h. Data represents means of three independent experiments ± SEM. *P < 0.05, **P < 0.01

As mentioned above, there might be a connection between SBF2-AS1 and miR-151a-3p. Increasing evidence shows that lncRNAs can function as ceRNAs for miRNAs or interact with RNA-binding proteins to regulate target gene expression [24, 25]. To further evaluate the molecular mechanism by which SBF2-AS1 promotes TMZ resistance in GBM cells, we firstly used FISH and subcellular fractionation to analyze its distribution. The results showed that SBF2-AS1 was primarily observed in the cytoplasm (Fig. 5a and Additional file 4 Figure S3A), indicating that SBF2-AS1 might function as a ceRNA to regulate target expression. To confirm that miR-151a-3p binds directly to SBF2-AS1, we set up a dual luciferase reporter assay. The observed miR-151a-3p-mediated suppression of luciferase activity was inhibited by mutation (MUT) of SBF2-AS1 (Fig. 5b). Moreover, RNA-binding protein immunoprecipitation (RIP) assay showed that in control GBM cells, the amount of lncSBF2-AS1 and miR-151a-3p immunoprecipitated with Ago2 was higher than respective IgG group, and knockdown of miR-151a-3p restrained the enrichment of SBF2-AS1 and miR-151a-3p in Ago2 precipitates (Fig. 5c and d). Then, we analyzed the correlation between lncSBF2-AS1 and miR-151a-3p levels in 20 pairs of recurrent GBM cancer tissues by using qRT-PCR. The result showed an inverse correlation between lncSBF2-AS1 and miR-151a-3p levels (Additional file 4 Figure S3B). Collectively, these data demonstrate that SBF2-AS1 sponges miR-151a-3p by acting as a ceRNA.

To investigate whether miR-151a-3p participated in SBF2-AS1-mediated apoptosis of GBM cells, miR-151a-3p was knocked down or overexpressed in SBF2-AS1-depleted GBM cells. Then the apoptosis of GBM cells in response to TMZ was determined. The results showed that overexpression of miR-151a-3p promoted the suppressive effect on TMZ resistance, which was induced by SBF2-AS1 knockdown in GBM cells, while inhibition of miR-151a-3p reversed the SBF2-AS1 knockdown-mediated apoptosis in GBM cells in response to TMZ (Fig. 5e and f). Together, these results showed an involvement of miR-151a-3p in lncSBF2-AS1-mediated TMZ resistance in GBM cells.

### LncSBF2-AS1 regulates DNA damage rapair through miR-151a-3p/XRCC4 axis

**Fig. 6:**
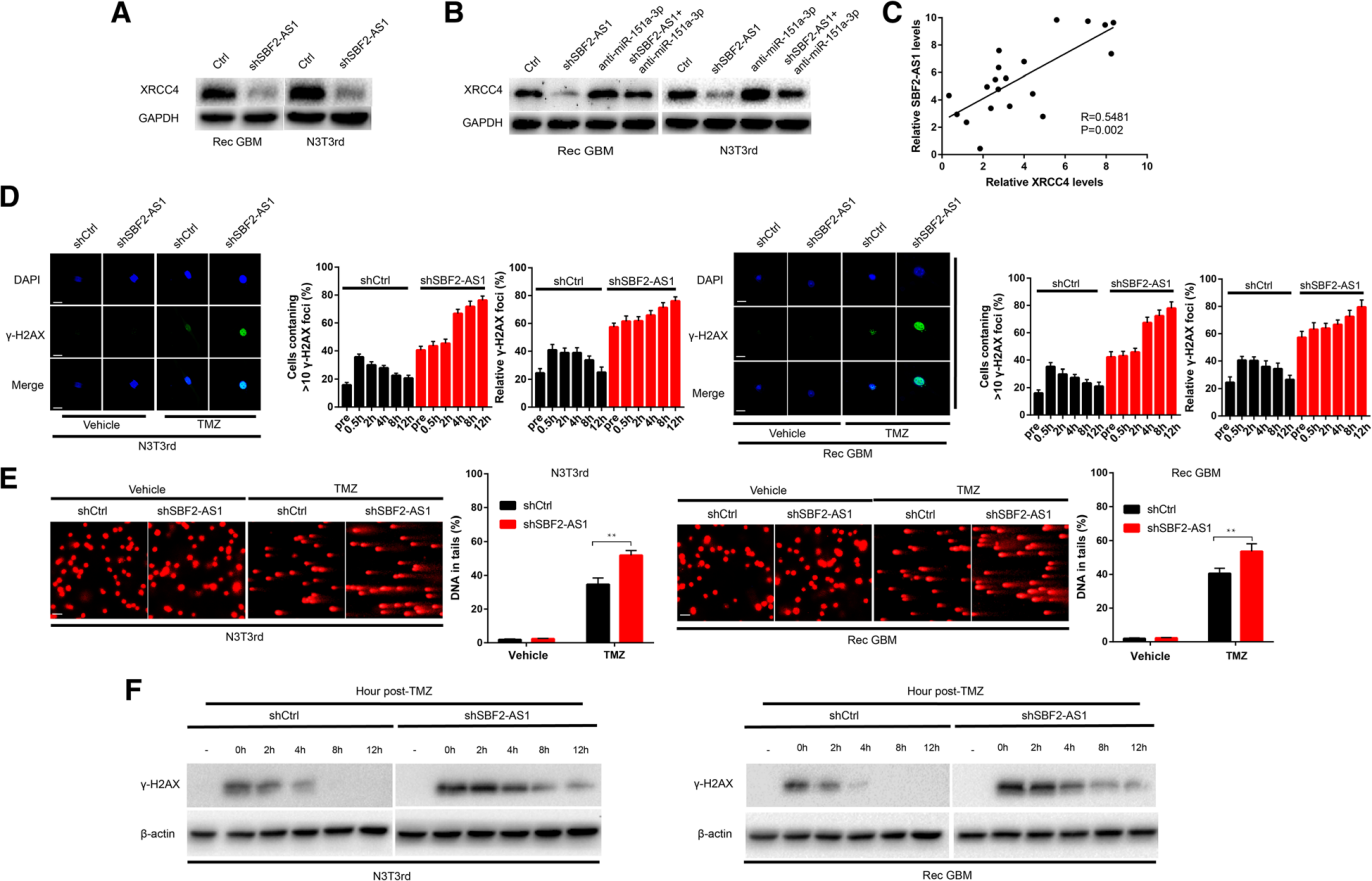
XRCC4 is indirectly regulated by lncSBF2-AS1, and SBF2-AS1 depletion induces a delay in DNA damage repair. **a** XRCC4 protein level in N3T3rd and Rec GBM cells following knockdown of lncSBF2-AS1. GAPDH was used as the loading control. **b** XRCC4 protein level in N3T3rd and Rec GBM cells following knockdown of lncSBF2-AS1 and/or inhibition of miR-151a-3p. GAPDH was used as the loading control. **c** Association analysis of the relationship between lncSBF2-AS1 and XRCC4 expression levels in 20 recurrent GBM tissues. **d** Immunofluorescence staining of γ-H2AX foci in shSBF2-AS1 or shctrl Rec GBM and N3T3rd cells 8 h after TMZ removal. Data represents means of three independent experiments ± SEM. **P < 0.01. Scale bar, 10 μm. **e** Comet assay of Rec GBM and N3T3rd cells transduced with shSBF2-AS1 or shctrl at the indicated time after TMZ withdrawal. Scale bar, 50 μm. **f** Western blot analysis of γ-H2AX expression in Rec GBM and N3T3rd cells transfected with shSBF2-AS1 or shctrl at different time points after TMZ treatment. β-actin was used as the loading control

As lncSBF2-AS1 can sponge miR-151a-3p and XRCC4 is a miR-151a-3p target gene [13], we determined whether lncSBF2-AS1 can regulate the expression of XRCC4 by sponging miR-151a-3p. We found knockdown of lncSBF2-AS1 also reduced XRCC4 protein levels in N3T3rd and recurrent GBM cells (Fig. 6a). To determine whether miR-151a-3p plays an important role between lncSBF2-AS1 and XRCC4, we co-transfected miR-151a-3p inhibitor and shSBF2-AS1 into recurrent GBM cells. Indeed, the suppression of XRCC4 protein levels induced by shSBF2-AS1 was effectively reversed by the miR-151a-3p inhibitor (Fig. 6b). Then, we analyzed the correlation between lncSBF2-AS1 and XRCC4 expression in 20 recurrent GBM cancer tissues. The result showed a positive correlation between lncSBF2-AS1 and XRCC4 expression, consistent with the existence of an lncSBF2-AS1-miR-151a-3p-XRCC4 regulatory axis (Fig. 6c). Together, these data suggested that lncSBF2-AS1 regulates the expression of XRCC4 by post-transcriptional modulation of miR-151a-3p.

Recent studies have reported that TMZ resistance can be promoted by DNA double-strand break (DSB) repair [26–28] and lncRNAs play an essential role in DSB [29, 30]. Thus, we wondered whether improved DSB repair is directly dependent on lncSBF2-AS1. A sensitive marker of DSBs, γ-H2AX [31], was assessed by confocal microscopy. We found that although the average number of γ-H2AX focis were slightly different in control shRNA (shctrl) and shSBF2-AS1 groups, the number of γ-H2AX focis in shSBF2-AS1 cells showed a significant increase within 8 h after TMZ removal, whereas shctrl cells had a constantly low number of γ-H2AX (Fig. 6d), even at 12 h. We next employed comet assay to survey DNA repair progression. The tail was still detectable in SBF2-AS1-depleted GBM cells 12 h after TMZ withdrawal. Meanwhile, the process of DSB clearance was completed within 12-h after TMZ removal in control cells (Fig. 6e). Furthermore, using Western blot assay, we found persistence of high expression level of γ-H2AX in GBM cells lacking SBF2-AS1, but not control cells 12 h after the end of TMZ treatment (Fig. 6f). These data showed that depletion of lncSBF2-AS1 delayed the repair process of TMZ-induced DNA damage.

### Intercellular transfer of lncSBF2-AS1 by exosomes spreads TMZ resistance in vitro

**Fig. 7:**
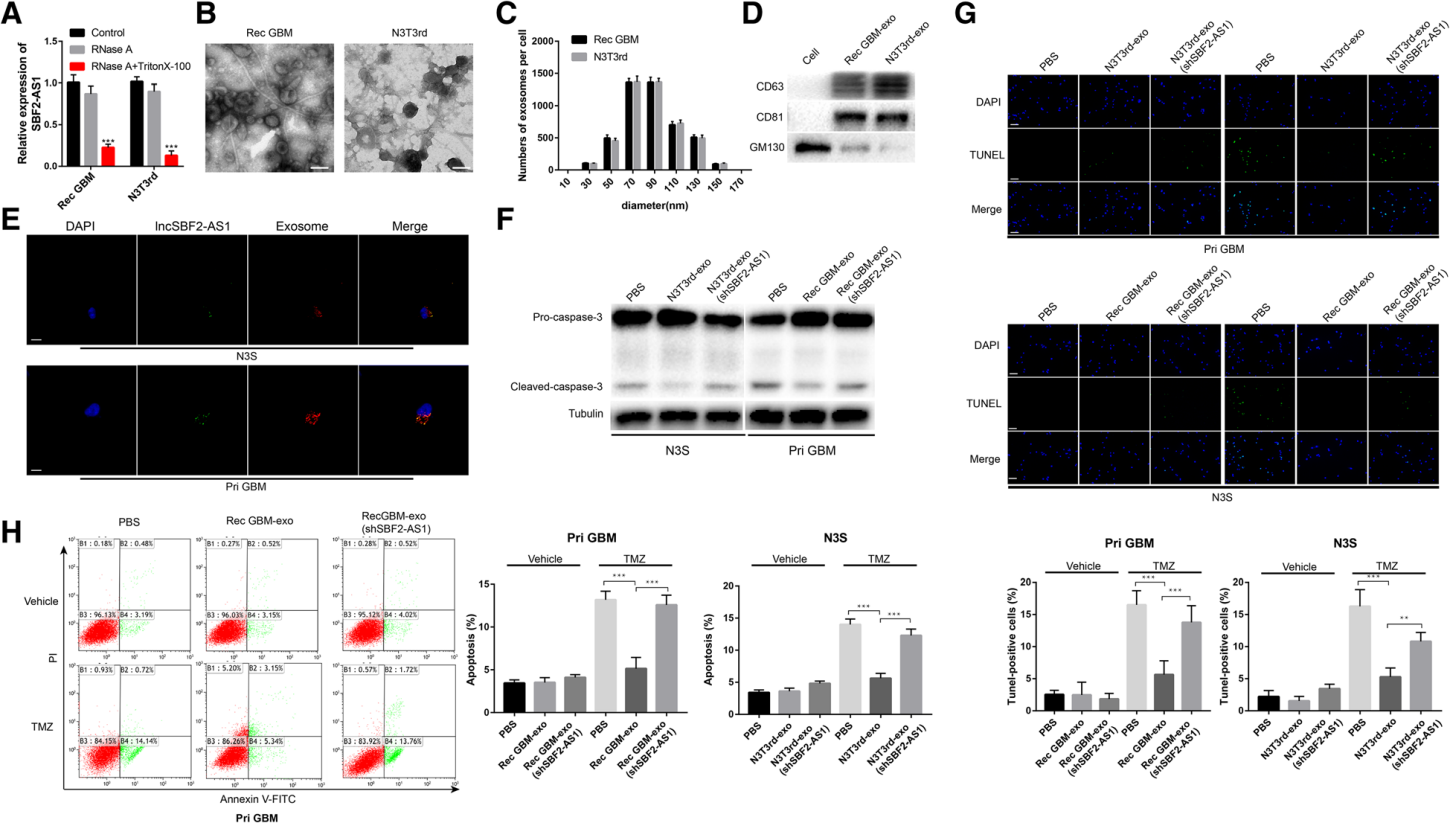
Exosomal transfer of lncSBF2-AS1 from chemoresistant cells to chemoresponsive cells and spread of TMZ resistance (**a**) The expression level of lncSBF2-AS1 in the culture medium of Rec GBM and N3T3rd cells treated with RNase (2 μg/ml) alone or combined with 0.1% Triton X-100 for 20 min. **b** Representative transmission electron microscopy (TEM) of exosomes secreted by TMZ-resistant cells. Scale bar, 100 nm. **c** The size distributions of exosomes derived from TMZ-resistant cells was analyzed by nanoparticle tracking assay. **d** Western blot analysis for exosomal marker CD63, CD81, and GM130 of exosomes derived from TMZ-resistant cells. Equal amount of these exosomes (500 ng) were used for Western blot analysis. **e** Exosomes were isolated from culture medium from Rec GBM and N3T3rd co-transfected with FAM-labelled SBF2-AS1 (green) and exosomal marker CD63 (red) and added to Pri GBM or N3S cell cultures. Confocal microscope was used to detect fluorescence signal in Pri GBM or N3S cells. Scale bar, 10 μm. **f** Western blot analysis of cleaved caspase-3 expression of exosome-treated chemoresponsive cells or untreated GBM cells in the presence of TMZ. Tubulin was used as the loading control. **g** TUNEL analysis of chemoresponsive GBM cells treated with TMZ-resistant exosomes in the presence of TMZ for 48 h. Data represents means of three independent experiments ± SEM. **P < 0.01, ***P < 0.001. Scale bar, 50 μm (**h**) Flow cytometry was used to measure the apoptosis of chemoresponsive GBM cells in the absence or presence of exosomes after exposure to 200 μM TMZ for 48 h. Data represents means of three independent experiments ± SEM. ***P < 0.001 (Rec GBM-exo: exosomes isolated from recurrent GBM cells; N3T3rd-exo: exosomes isolated from N3T3rd cells; Rec GBM-exo (shSBF2-AS1): exosomes isolated from shSBF2-AS1 recurrent GBM cells; N3T3rd-exo (shSBF2-AS1): exosomes isolated from shSBF2-AS1 N3T3rd cells)

Cell-secreted exosomes and their contents can be internalized by surrounding cells [32, 33]. Nucleotide is one of the inclusions of exosomes [34] and lncRNAs transferred by exosomes promote drug resistance [35, 36]. To investigate the manner of SBF2-AS1 intercellular delivery, the level of SBF2-AS1 was analyzed by qRT-PCR. We found that the level of SBF2-AS1 in the culture medium showed only a slight change upon RNase treatment but markedly decreased after treated with RNase and Triton X-100 synchronously (Fig. 7a), indicating that extracellular SBF2-AS1 was mainly packed in membranes. Then we isolated exosomes derived from TMZ-resistant glioma cells and parental cells. The characteristics were analyzed, and quantification was done using transmission electron microscopy (TEM), nanoparticle tracking analysis (NTA) and Western blot analysis. A typical lipid bilayer membrane was observed, and the size of exosomes were confirmed by TEM and NTA (Fig. 7b and c). A Western blot analysis showed the proteins CD63 and CD81 were positively expressed in exosomes and GM130 was mainly present in the cell lysate [37] (Fig. 7d).

Next, we determined whether the exosomes of TMZ-resistant cells (Rec GBM and N3T3rd cells) transferred SBF2-AS1 to parental cells (Pri GBM and N3S cells). Rec GBM and N3T3rd cells were co-transfected with fluorescein amidite (FAM)-tagged SBF2-AS1 and exosomal marker CD63. The exosomes were isolated from culture medium collected from Rec GBM and N3T3rd cells. Parental cells were treated with purified exosomes. SBF2-AS1 wrapped by exosomes and internalization of exosomes were observed by confocal microscopy (Fig. 7e). To further confirm the effect of exosomal lncSBF2-AS1, we knocked down SBF2-AS1 in TMZ-resistant cells and the level of lncSBF2-AS1 in exosomes echoed (Additional file 5 Figure S4A). Then the exosomes were used to treat parental cells and we found that the apoptosis of parental cells, treated with Rec GBM and N3T3rd exosomes showed a significant decrease after TMZ treatment, however, knockdown of exosomal SBF2-AS1 reversed the apoptosis of GBM cells (Fig. 7f, g and h). Meanwhile, the lncSBF2-AS1 transfer of TMZ-resistant exosomes downregulated XRCC4 expression in Pri GBM and N3S cells (Additional file 5 Figure S4B), and exosomal lncSBF2-AS1 accelerated the DNA damage repair in GBM recipient cells after TMZ treatment (Additional file 5 Figure S4C and D). Thus, our data indicated that exosomal lncSBF2-AS1 could enhance chemoresistance to TMZ in vitro.

### Exosomal lncSBF2-AS1 spreads TMZ resistance in vivo

**Fig. 8:**
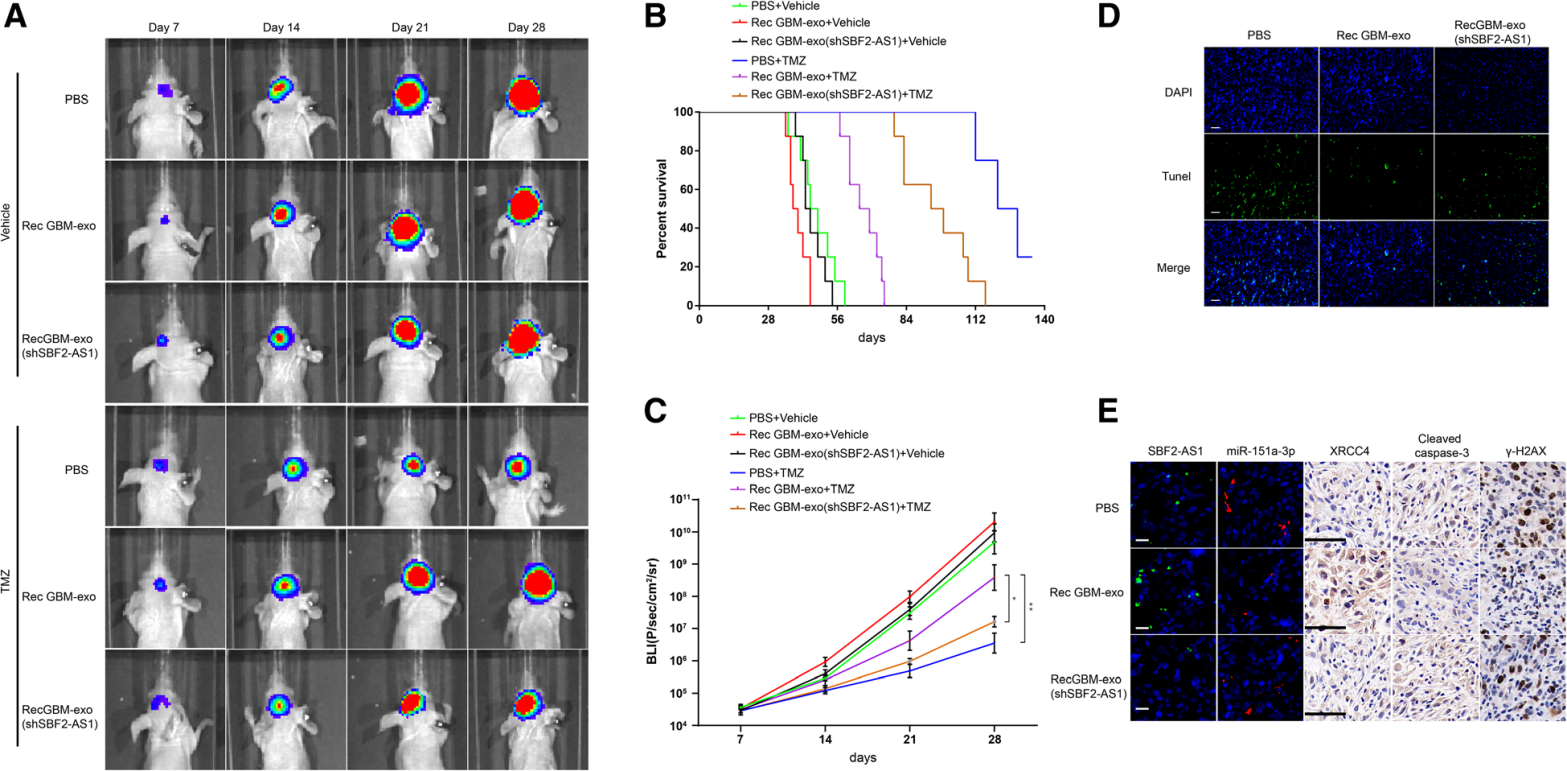
Exosomal transfer of lncSBF2-AS1 enhances chemoresistance to TMZ in vivo. **a** Representative bioluminescence images of intracranial xenografts derived from Pri GBM, which were pre-incubated with PBS or 50 μg/ml Rec GBM-exo or Rec GBM-exo (shSBF2-AS1) for 2 days. **b** Survival curves of nude mice derived from Pri GBM cells indicated above in the absence or presence of TMZ. **c** Bioluminescence was measured in tumors from six groups. Data represents means of three independent experiments ± SEM. *P < 0.05, **P < 0.01. **d** TUNEL staining of intracranial xenografts derived from Pri GBM cells indicated above in response to TMZ. Scale bar, 50 μm (**e**) The expression of lncSBF2-AS1, miR-151a-3p, XRCC4, γ-H2AX, and cleaved caspase-3 were analyzed by FISH or IHC analysis on intracranial xenografts derived from Pri GBM cells indicated above in response to TMZ. Scale bar, 50 μm (left, FISH) and 50 μm (right, IHC)

To investigate whether exosomal lncSBF2-AS1 is necessary for TMZ resistance in vivo, mice were orthotopically injected with 2.5 × 10^5^ luciferase-labeled Pri GBM cells following pre-incubation with PBS, Rec GBM-exo or Rec GBM-exo (shSBF2-AS1) for 2 days. The tumor-bearing mice were exposed to DMSO or TMZ (66 mg/kg/day) for 5 days each week for three cycles and analyzed by bioluminescence imaging. Control xenografts showed tumor progression. We found that xenografts carrying Rec exosomes treated Pri GBM cells displayed a significant advance of tumor growth in response to TMZ. Meanwhile, SBF2-AS1-depleted Rec exosomes treated Pri GBM cells demonstrated a regression in tumor progression after TMZ treatment (Fig. 8a). The findings were also confirmed by the survival curves (Fig. 8b), bioluminescence assay (Fig. 8c) and TUNEL staining (Fig. 8d). Moreover, we performed FISH and IHC on xenograft tissues receiving TMZ therapy. Tumors with high SBF2-AS1 levels (Rec-exo treated) tended to express lower miR-151a-3p and lower protein levels of cleaved caspase-3 and γ-H2AX and higher protein level of XRCC4 (Fig. 8e). Higher protein levels of cleaved caspase-3 and γ-H2AX and higher miR-151a-3p level together with lower XRCC4 level was observed in low SBF2-AS1 groups (PBS treated and Rec GBM-exo (shSBF2-AS1) treated) (Fig. 8e). These data indicate that exosomal lncSBF2-AS1 promoted development of acquired resistance to TMZ in vivo*.*

### Circulating exosomal lncSBF2-AS1 may act AS a promising diagnostic biomarker for TMZ-resistant GBM patients

**Fig. 9:**
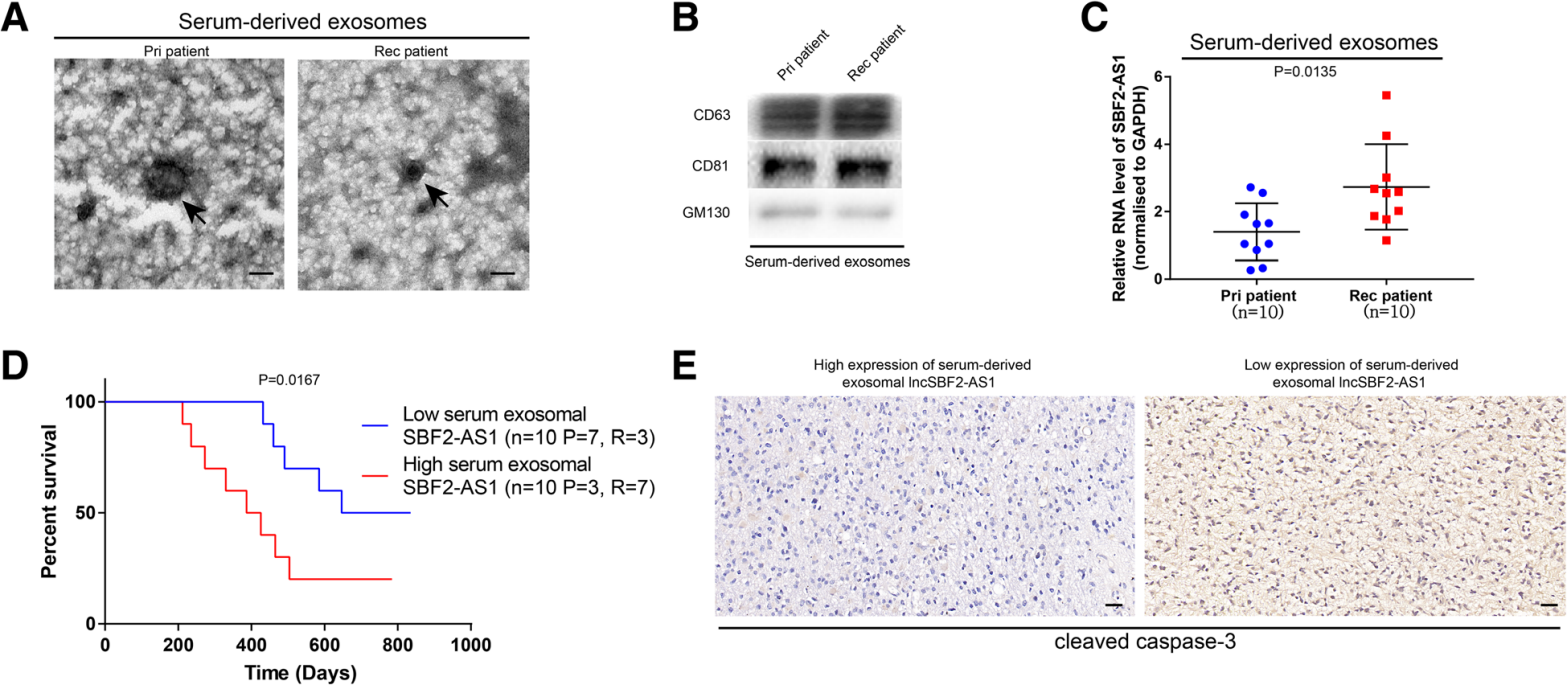
Expression of serum exosomal lncSBF2-AS1 is associated with TMZ resistance in GBM patients. **a** Representative transmission electron microscopy (TEM) of serum-derived exosomes from recurrent GBM patients and primary GBM patients. Scale bar, 100 nm. **b** Western blot assay for exosomal markers CD63, CD81 and GM130 of serum-derived exosomes from recurrent GBM patients and primary GBM patients. **c** qRT-PCR analysis of lncSBF2-AS1 expression in serum-derived exosomes from recurrent GBM patients and primary GBM patients. Data represents means of three independent experiments ± SEM. **d** Survival curves of 20 GBM patients who received TMZ treatment, with low (*n* = 10) and high (*n* = 10) serum exosomal lncSBF2-AS1 levels. The number of primary patients (P) and recurrent patients (R) per survival group were indicated. **e** Immunohistochemical staining of cleaved caspase-3 in GBM samples with high exosomal lncSBF2-AS1 levels or low exosomal lncSBF2-AS1 levels. Scale bar, 50 μm

Exosomal RNAs can be transferred to neighboring cells or cells in other organs through the circulation system [38]. Furthermore, exosomal lncRNA has been considered as a potential biomarker of tumor chemoresistance [39]. Thus, exosomal lncSBF2-AS1 secreted by recurrent GBM cells may be detected in circulation. To confirm our assumption, we extracted serum exosomes collected from recurrent GBM patients and compared them with primary GBM patients (patient information are listed in Additional file 6: Table S3). Purified exosomes were authenticated by TEM analysis and Western blotting analysis (Fig. 9a and b). qRT-PCR assay was performed, and we found that exosomal lncSBF2-AS1 was enriched in the serum of recurrent GBM patients instead of primary GBM patients (Fig. 9c). Recurrent GBM patients with higher serum exosomal SBF2-AS1 levels had worse prognosis, forecasting a poor response to TMZ treatment (Fig. 9d). IHC staining was performed to evaluate the correlation between exosomal lncSBF2-AS1 and apoptosis marker cleaved caspase-3 in recurrent GBM patients. The results showed that exosomal lncSBF2-AS1 levels in the serum of recurrent GBM patients were positively correlated with cleaved caspase-3 expression (Fig. 9e). Altogether, our data indicates that exosomal lncSBF2-AS1 may serve as a promising diagnostic biomarker for recurrent GBM patients.

## Discussion

The therapeutic options for glioblastoma multiforme patients are limited by TMZ resistance. Thus, it is necessary to investigate the biological basis of TMZ resistance and identify novel targets for the prevention and therapy of resistance to TMZ. Growing evidence has emphasized the association of lncRNAs with tumor chemoresistance and the possibility that lncRNAs function as biomarkers [39–41]. Aberrantly expressed lncRNAs are crucial regulatory factors in GBM [42–44]. To identify the potential biomarkers for TMZ treatment, we focused on exosomal lncRNAs, and investigated the functional correlation between TMZ resistance and specific lncRNAs in GBM. In the present study, we identified an uncharacterized lncRNA, lncSBF2-AS1, which may function as an oncogene in GBM by promoting cell TMZ-resistance, and inhibition of lncSBF2-AS1 might serve as a promising clinical therapeutic approach in GBM treatment. Meanwhile, lncSBF2-AS1 may function as a hopeful biomarker for reaction to chemotherapy.

LncRNAs have been further reported as playing critical roles in cancer progression [45, 46] especially chemoresistance [47]. Combined with our previous report [13], we identified lncRNA SBF2-AS1 as a potential lncRNA, which is necessary for enhancing TMZ resistance. LncRNA SFB2-AS1 (ENSG00000246273) has been rarely reported. It was first described as an oncogene in non-small cell lung cancer [48] and has been recently reported as a key molecule in GBM angiogenesis [12]. Herein, we reported a novel function of SBF2-AS1 in TMZ resistance. SBF2-AS1 is activated by the transcription factor ZEB1 and we detected that SBF2-AS1 was upregulated in GBM cells and tissues derived from recurrent GBM patients who were resistant to TMZ treatment. Moreover, ectopic expression of SBF2-AS1 in parental GBM cells facilitated acquirement of TMZ resistance, however, depletion of SBF2-AS1, sensitized TMZ-refractory GBM cells to TMZ in vitro and in vivo. These results were further supported by the detection that xenografts expressing low levels of SBF2-AS1 exhibited markedly better survival after TMZ treatment compared with xenografts expressing high levels of SBF2-AS1. Thus, our data shed insight on the importance of SBF2-AS1 as a potential prognostic and predictive marker of response to TMZ.

To futher understand the role of lncSBF2-AS1 in GBM, we localized its expression in GBM cells and found that lncSBF2-AS1 mainly expressed in the cytoplasm, which indicates lncSBF2-AS1 may function as miRNA sponges. Competing endogenous RNA (ceRNA), a novel and widespread interaction network, where lncRNAs could regulate miRNA through binding and titrating them off their binding sites on protein coding messengers [19, 49]. For example, LncRNA FER1L4 functions as a ceRNA to modulate PTEN expression through regulation of miR-106a-5p in gastric cancer [50]. LncRNA CASC2 acts as a sponge for miR-21 to sensitize cervical cancer to cisplatin [51]. LncRNA KCNQ1OT1 regulates cisplatin resistance by acting as a ceRNA to modulate the expression of miR-211-5p in tongue cancer [52]. Combined with our previous results and bioinformatic analyses, we used RIP analysis and luciferase reporter assays to verify if miR-151a-3p acts as a direct target for both SBF2-AS1 and XRCC4 genes and is responsible for SBF2-AS1-mediated TMZ resistance. Our findings discovered the significance of the interaction between SBF2-AS1 and miR-151a-3p in GBM TMZ resistance and that SBF2-AS1 acts an oncogene partly by sponging miR-151a-3p in GBM cells.

Acceleration of DNA DSB repair can enhance TMZ chemoresistance in GBM cells, and increase the survival of GBM cells under TMZ treatment [26, 53], resulting in tumor recurrence. XRCC4, a major factor for DSB repair, has recently been verified to be associated with TMZ resistance [54–56]. Our previous study has reported miR-151a-3p can regulate TMZ resistance in GBM cells by targeting XRCC4. However, function as the upstream of miR-151a-3p, the effect of lncSBF2-AS1 on DNA damage repair is still unclear.. In the present study, we tried to collect direct evidence to demonstrate that depletion of SBF2-AS1 markedly reduced XRCC4 levels, retarded the process of DSB repair, and induced cells to become sensitive to TMZ.

Zinc finger E-box binding homeobox 1 (ZEB1) is the key factor of Epithelial-mesenchymal transition (EMT) and cancer cells undergoing EMT show more chemoresistance [23, 57, 58]. In recent study, ZEB1 can regulate therapy resistance in lung adenocarcinoma [59], hepatocarcinoma [60] and ovarian cancer [61]. In the present study, we found ZEB1 can binding the promoter region of lncSBF2-AS1 and regulate the expression level of lncSBF2-AS1. Meanwhile, overexpression or depletion of ZEB1 represented the changes of TMZ-resistance in GBM. Above all, our data found the regulatory mechanism of lncSBF2-AS1 and indicated the important role of ZEB1 on chemoresistance of GBM.

A growing number of evidences suggest exosomes can protect lncRNAs from degradation in the circulation and exosomal lncRNAs could be used to monitor cancer at an early stage [62, 63]. Exosomes are nano-sized vesicles that exhibit vesicular–like characteristics and fuse with plasma membranes of various cell types [64]. Increasing reports have shown the unique characteristics of exosomes, including their ability to package specific lncRNAs, circRNAs and miRNAs with stability and easy detection in the circulatory system [65]. They have been confirmed as intercellular messengers that transfer cellular components, including lncRNAs [66]. In our study, we found that lncSBF2-AS1 can be enclosed into exosomes. Parental GBM cells treated with TMZ-resistant exosomes exhibited a resistant phenotype. Meanwhile, knockdown of exosomal lncSBF2-AS1 can partially reverse chemoresistance of parental cells. In addition, the in vivo experiments also demonstrated exosomal lncSBF2-AS1 can spread TMZ resistance.

Based on the functional observations, exosomal SBF2-AS1 level was determined in clinical serum samples. Repeated attempts have been made to use lncRNAs in circulation as biomarkers [67]. Nevertheless, these potential predictors are always in relatively low abundance and easily degradable. LncRNAs are enriched and steady in the circulatory exosome system. Thus, the levels of exosomal lncRNAs can help in the prognosis of several cancers, and thus, can be used as cancer biomarkers [35, 39]. Our result showing that SBF2-AS1 levels in the serum of recurrent GBM patients who were under TMZ treatment were higher as compared to normal subjects. These data provide novel evidence that circulating exosomal SBF2-AS1 may serve as a potential predictor for TMZ-resistant GBMs.

## Conclusion

Collectively, our study demonstrated that exosome-mediated transfer of lncRNA SBF2-AS1 spreads TMZ resistance in GBM cells. Exosomal SBF2-AS1 levels in human serum may function as a potential diagnostic biomarker in GBM patients, optimizing the clinical benefits of TMZ therapy and have important significance in the development of treatment strategies for TMZ-resistant GBM patients.

## Additional files

Additional file 1: Supplementary materials and methods. (DOCX 21 kb)

Additional file 2: **Figure S1.** A Expressions of lncRNAs were analyzed by qRT-PCR in N3 cell. The data represent the mean±SEM from three independent expriments. *P < 0.05, **P < 0.01. B The luciferase reporter plasmid carrying miR-151a-3p was co-transfected into HEK293T cell with the 5 various lncRNA-coding plasmids. The data represent the mean±SEM from three independent expriments. *P < 0.05, **P < 0.01. C Caspase-3 activity in N3 cells transfected with the 5 various lncRNA siRNA after TMZ treatment. The data represent the mean±SEM from three independent expriments. *P < 0.05, **P < 0.01. D LncSBF2-AS1 expression was analyzed by qRT-PCR in normal human astrocytes and five GBM cell lines. Data represents means of three independent experiments ± SEM. *P < 0.05, **P < 0.01, ***P < 0.001 E Relative expression of lncSBF2-AS1 in glioblastoma (GBM) tissues compared with low grade glioma (LGG) tissues analyzed using TCGA data. F Kaplan-Meier overall survival according to lncSBF2-AS1 expression levels. (TIF 182 kb)

Additional file 3: **Figure S2.** A The luciferase reporter plasmids carrying lncSBF2-AS1 promoter region were co-transfection into HEK293T cells with five transcription factor (NRF1, KLF5, GATA2, ZEB1, NFκB) plasmids, respectively. Relative luciferase activity in HEK293T cells were determined. The data represent the mean±SEM from three independent expriments. **P < 0.01, ***P < 0.001. B Western blot analysis of ZEB1 expression in Rec GBM, Pri GBM, U87T3rd, U87S, N3T3rd and N3S cells. β-actin was used as the loading control. (TIF 183 kb)

Additional file 4: **Figure S3.** A qRT-PCR analysis of RNA expression level in nuclear and cytoplasm of GBM cells. U6 (nuclear retained) and GAPDH (exported to cytoplasm) were used as controls. B Association analysis of relationship between lncSBF2-AS1 and miR-151a-3p expression, in 20 recurrent GBM tissues. (TIF 154 kb)

Additional file 5: **Figure S4.** A qRT-PCR analysis of lncSBF2-AS1 expression level in exosomes isolated from N3T3rd and Rec GBM cells, which were transfected shCtrl or shSBF2-AS1. The data represent the mean±SEM from three independent expriments. **P < 0.01 B Western blot assay for XRCC4 in Pri GBM and N3S cells treated with PBS, Rec GBM-exo/N3T3rd-exo or Rec GBM-exo (shSBF2-AS1)/N3T3rd-exo (shSBF2-AS1). GAPDH was used as the loading control. C Immunofluorescence staining of γ-H2AX foci in Rec GBM or N3T3rd cells which incubation with indicated exosomes for 2-day at 12h after TMZ exposure (200μM). Scale bar, 10μm. D Comet assay of Pri GBM and N3S cells treated with indicated exosomes at the indicated time after TMZ withdrawal. Data are means of three independent experiments±SEM. **P < 0.01. Scale bar, 50μm. (TIF 1722 kb)

Additional file 6: **Table S1.** Twenty GBM patients and treatment characteristic. **Table S2.** Primers for qRT-PCR and siRNA target squence. **Table S3.** Clinicopathological features of 20 GBM patients and treatment characteristic. **Table S4.** Clinicopathological features of GBM patients in TCGA database. (DOCX 24 kb)

## Abbreviations

Ago2: Argonaute2
DMEM: Dulbecco’s modified Eagle’s medium
FISH: Fluorescence in situ hybridization
GAPDH: Glyceraldehyde 3-phosphate dehydrogenase
GBM: Glioblastoma multiforme
IHC: Immunochemistry
TMZ: Temozolomide
XRCC4: X-ray repair cross complementing 4
ZEB1: Zinc finger E-box binding homeobox 1
