# Supplementary material for: Exosomal transfer of long non-coding RNA SBF2-AS1 enhances chemoresistance to temozolomide in glioblastoma
Source: J Exp Clin Cancer Res. 2019 Apr 16;38:166. doi: 10.1186/s13046-019-1139-6 (PMC6469146; doi:10.1186/s13046-019-1139-6)
Supplement: Supplementary file 1 — Supplementary materials and methods. (DOCX 21 kb) [file 13046_2019_1139_MOESM1_ESM.docx]

**Supplementary materials and methods**

***Clinical specimens***

Glioblastoma (GBM) lncRNA expression and survival data were downloaded from The Cancer Genome Atlas (TCGA) dataset (<http://cancergenome.nih.gov>). We obtained 20 primary and their corresponding recurrent GBM specimens (each pair was from the same patient) from patients who were under TMZ treatment from the Department of Neurosurgery, the First Affiliated Hospital of Nanjing Medical University, Nanjing, China ( Patient information are listed in Supplementary table S1). Twenty human serum samples from GBM patients were also collected from the same department (the First Affiliated Hospital of Nanjing Medical University, Nanjing, China), patient information are listed in Supplementary table S3.

***Exosome isolation***

Exosomes were extracted from GBM cell culture through standard centrifugation steps as previously described ([1](#_ENREF_1)). The medium was replaced with DMEM medium supplemented with 10% exosome-depleted FBS when cells had grown to 70%-80% confluence. After 48-hours, the cell supernatant was preliminarily centrifuged at 3000 × g for 30 min at 4℃ to remove cellular debris/dead cells. Then the resulting supernatant was processed at 100,000 × g for 70 min at 4℃. For serum exosome isolation, serum was centrifuged within 2 hours after collection at 1600 g for 10 min (at room temperature) and the supernatant was collected. Then a second centrifugation was performed at 12000 × g for 10 min at 4℃. Serum exosomes were isolated using ExoQuick precipitation kit (SBI, System Biosciences, Mountain View, CA) according to manufacturer’s instructions.

***RNA extraction and quantitative reverse transcription (qRT) -PCR assays***

Extraction of total RNA from tissues and cultured cells was performed using Trizol (Invitrogen) according to the manufacturer’s protocol. Exosomal RNA was extracted by using the commercial miRNeasy serum/Plasma kit (QIAGEN, Waltham, MA). RNA was reverse transcribed using the PrimeScript RT reagent Kit (TaKara, Nanjing, China) and then RT-PCR analyses were performed with Gotaq® Green Master Mix (TaKara, Nanjing, China). The results were normalized with GAPDH. Primers for lncRNAs (LOC101593348, RP11-111F5.4, LINC00271, PSMD5-AS1, XLOC_003734, RP11-348P10.2, RP11-967K21.1, CTC-480C2.1, SBF2-AS1 and ARHGEF26-AS1) and internal controls (U6, β-actin and GAPDH), were purchased from Ribobio, Guangzhou, China. Primers are shown in Supplementary Table S2. Quantitative reverse transcription-PCR was carried out using ABI 7500 real-time PCR system (Applied Biosystems, Foster City, CA, USA). Fold change was determined as 2^-△△Ct^ in gene expression.

***Plasmid construction, transfection, and stable cell establishment***

For stable transfection, the lentivirus carrying lncSBF2-AS1 or negative control (Lv-NC) was packaged in GBM cells using the lentiviral packaging kit (Genechem Shanghai, China). Meanwhile, shSBF2-AS1 or negative control (shCtrl) lentiviral particles were purchased from Genechem (Shanghai, China). A172, Rec GBM and N3T3rd cells were used to establish stable cell lines and selected with puromycin at 48h after injection. For transient transfection, ZEB1 plasmid, ZEB1 siRNA, XRCC4 siRNA, miR-151a-3p mimics and miR-151a-3p inhibitor were purchased from Genechem (Shanghai, China) and transfected into cells using the method described previously ([2](#_ENREF_2)). GBM cells were transfected with oligonucleotides using Lipofectamine 2000 (Invitrogen, USA).

***Immunofluorescent staining***

Cells were fixed in 4% paraformaldehyde for 15 min and washed with PBS. Then 0.25% Triton X-100 (dissolved in PBS) was used to permeabilize the fixed cells. The cells were treated with 1% bovine serum albumin for 20 min and then incubated with primary antibody on a shaker at room temperature for 1 h. After being washed with PBS, the cells were incubated with goat anti-rabbit IgG secondary antibodies (FITC Green goat anti-rabbit, Molecular Probes, USA) on a shaker at room temperature for 1 h, followed by another PBS washing. The nucleic acids were detected with anti-fading mounting medium containing 4,6-diamidino-2-phenylindole (DAPI). After incubation in cytoskeleton buffer and stripping buffer on ice, the cells were fixed and processed as described before. The images were visualized with a Nikon ECLIPSE E800 fluorescence microscope.

***Luciferase assay***

Human lncSBF2-AS1 promoter region was amplified using PCR and inserted into pGL3-Basic luciferase reporter vector (Promega). HEK293T cells were co-transfected with luciferase reporter comprising SBF2-AS1 promoter region and empty vector or NRF1, KLF5, GATA2, ZEB1, NFκB plasmid (Genechem). HEK293T cells were co-transfected with luciferase reporter comprising miR-151a-3p (constructed by Genechem) and empty vector or RP11-111F5.4, ARHGEF26-AS1, PSMD5-AS1, RP11-967K21.1, SBF2-AS1 lncRNAs. N3 and recurrent GBM cells were co-transfected with wild type (WT) or mutant (MUT) SBF2-AS1 (constructed by Genechem) and miR-151a-3p. All luciferase assays were analyzed after 48h of transfection using Dual-Luciferase Kit (Promega). The luciferase activities were measured using the Dual Luciferase Reporter Assay System (Promega) and normalized to Renilla luciferase.

***Subcutaneous and orthotopic xenograft studies***

All experimental mice were purchased from the Experimental Animal Center of Nanjing Medical University. For subcutaneous xenograft studies, 1 × 10^7^ GBM cells were injected into 6-week-old male BALB/c nude mice and calipers were used to measure tumor length (L) and width (W). The formula, V= (L×W^2^) × 0.5 was used to calculated tumor volume (V). TMZ or the vehicle (DMSO) were orally gavaged to mice when tumor volume reached 55 mm^3^. For each cycle, 66 mg/kg/day TMZ was given to mice for 5 days per week. Mice were euthanized after the TMZ treatment was completed and the growth of subcutaneous xenograft tumor was examined. For patient-specific orthotopic xenograft studies, 2.5 × 10^5^ recurrent GBM cells (pretreated with lentivirus SBF2-AS1 or negative control sequences) were intracranially injected into nude mice via a stereotactic instrument. TMZ was given to tumor-bearing mice by oral gavage at 1 week (66 mg/kg per day for 5 days). Bioluminescence imaging (IVIS Spectrum, PerkinElmer, USA) was used to confirm tumor formation and tumor growth was measured each week.

**References**

1. Théry C, Amigorena S, Raposo G, Clayton A. Isolation and characterization of exosomes from cell culture supernatants and biological fluids. Curr Protoc Cell Biol. 2006;Chapter 3:Unit 3.22.

2. Zeng A, Wei Z, Yan W, Yin J, Huang X, Zhou X, et al. Exosomal transfer of miR-151a enhances chemosensitivity to temozolomide in drug-resistant glioblastoma. Cancer Lett. 2018;436:10-21.
