# Supplementary figures and images for: Exosomal transfer of long non-coding RNA SBF2-AS1 enhances chemoresistance to temozolomide in glioblastoma
Source: J Exp Clin Cancer Res. 2019 Apr 16;38:166. doi: 10.1186/s13046-019-1139-6 (PMC6469146; doi:10.1186/s13046-019-1139-6)

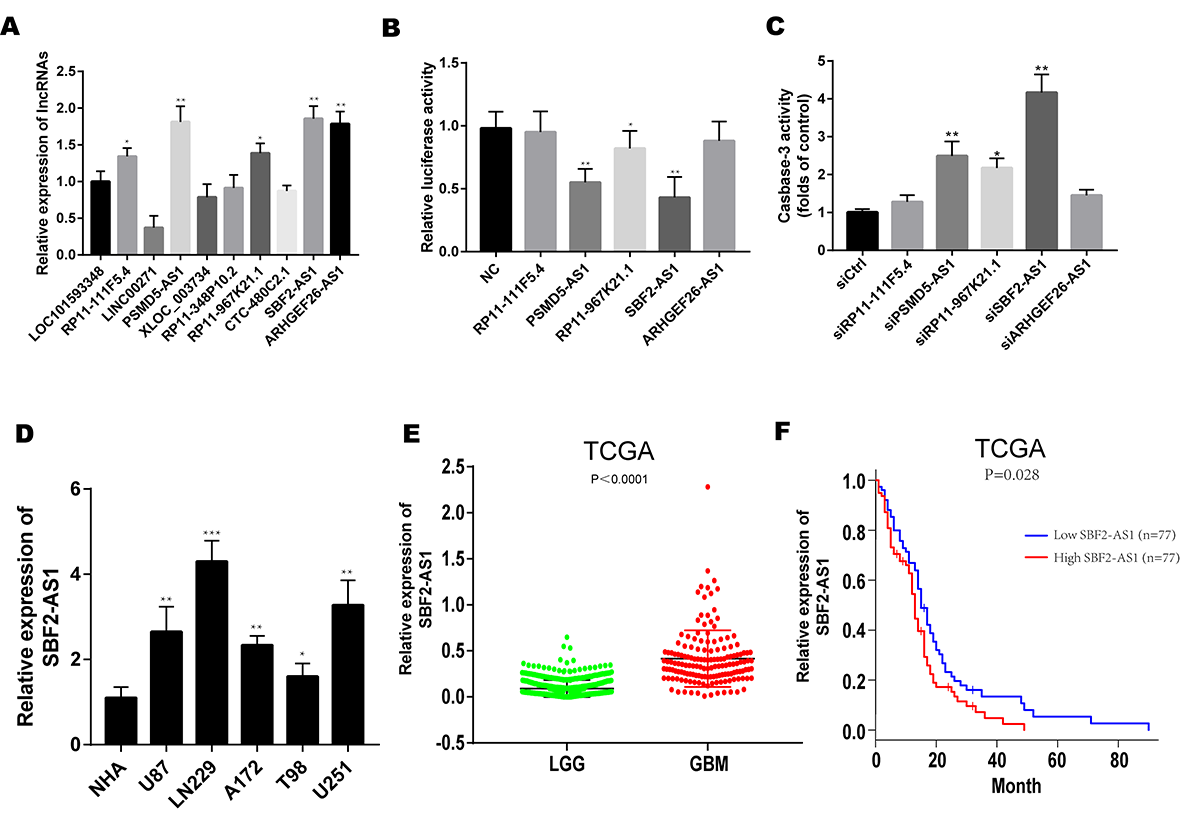

Supplement: Supplementary file 2 — Figure S1. A Expressions of lncRNAs were analyzed by qRT-PCR in N3 cell. The data represent the mean±SEM from three independent expriments. *P < 0.05, **P < 0.01. B The luciferase reporter plasmid carrying miR-151a-3p was co-transfected into HEK293T cell with the 5 various lncRNA-coding plasmids. The data represent the mean±SEM from three independent expriments. *P < 0.05, **P < 0.01. C Caspase-3 activity in N3 cells transfected with the 5 various lncRNA siRNA after TMZ treatment. The data represent the mean±SEM from three independent expriments. *P < 0.05, **P < 0.01. D LncSBF2-AS1 expression was analyzed by qRT-PCR in normal human astrocytes and five GBM cell lines. Data represents means of three independent experiments ± SEM. *P < 0.05, **P < 0.01, ***P < 0.001 E Relative expression of lncSBF2-AS1 in glioblastoma (GBM) tissues compared with low grade glioma (LGG) tissues analyzed using TCGA data. F Kaplan-Meier overall survival according to lncSBF2-AS1 expression levels. (TIF 182 kb) [file 13046_2019_1139_MOESM2_ESM.tif]

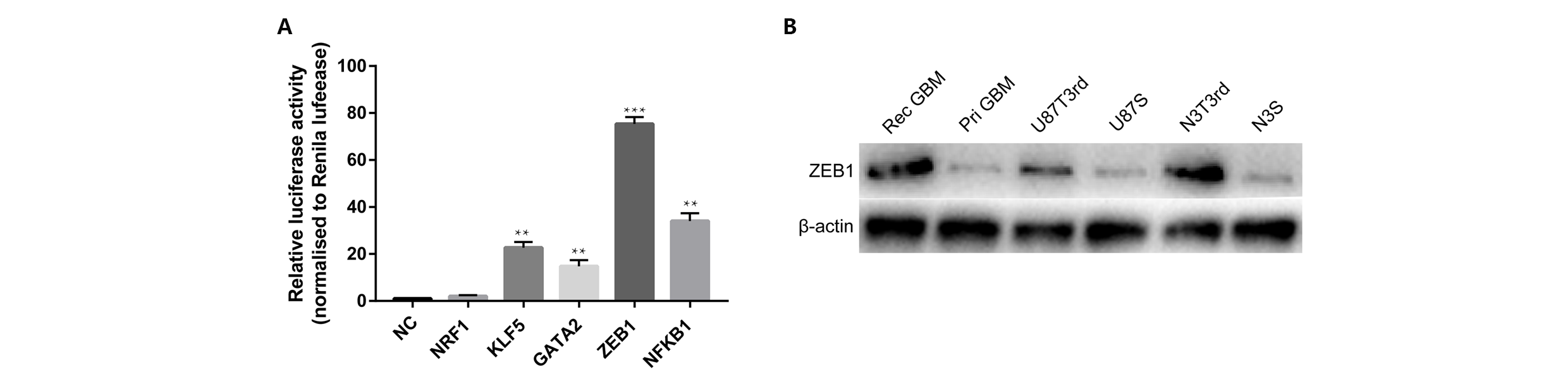

Supplement: Supplementary file 3 — Figure S2. A The luciferase reporter plasmids carrying lncSBF2-AS1 promoter region were co-transfection into HEK293T cells with five transcription factor (NRF1, KLF5, GATA2, ZEB1, NFκB) plasmids, respectively. Relative luciferase activity in HEK293T cells were determined. The data represent the mean±SEM from three independent expriments. **P < 0.01, ***P < 0.001. B Western blot analysis of ZEB1 expression in Rec GBM, Pri GBM, U87T3rd, U87S, N3T3rd and N3S cells. β-actin was used as the loading control. (TIF 183 kb) [file 13046_2019_1139_MOESM3_ESM.tif]

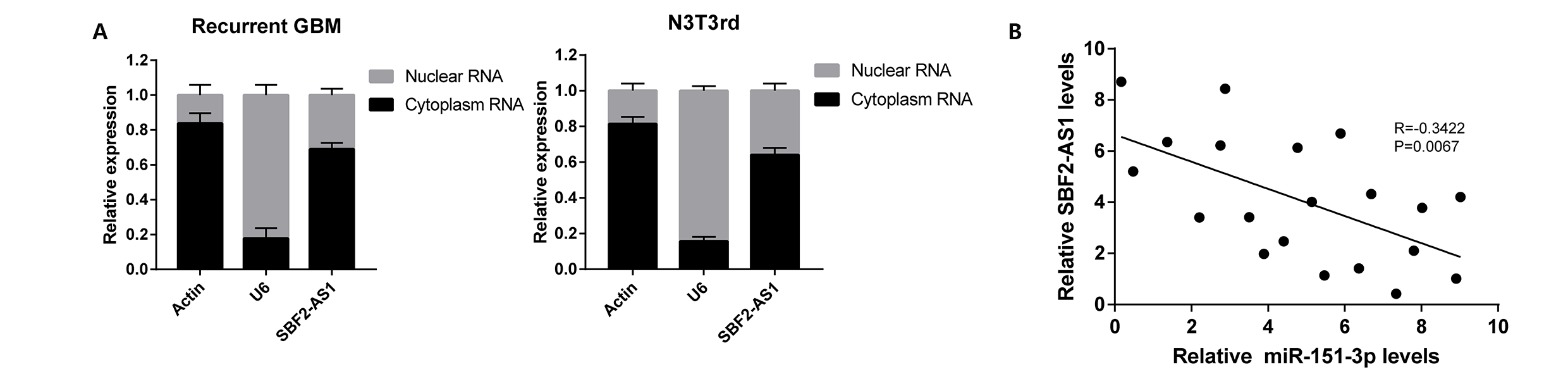

Supplement: Supplementary file 4 — Figure S3. A qRT-PCR analysis of RNA expression level in nuclear and cytoplasm of GBM cells. U6 (nuclear retained) and GAPDH (exported to cytoplasm) were used as controls. B Association analysis of relationship between lncSBF2-AS1 and miR-151a-3p expression, in 20 recurrent GBM tissues. (TIF 154 kb) [file 13046_2019_1139_MOESM4_ESM.tif]

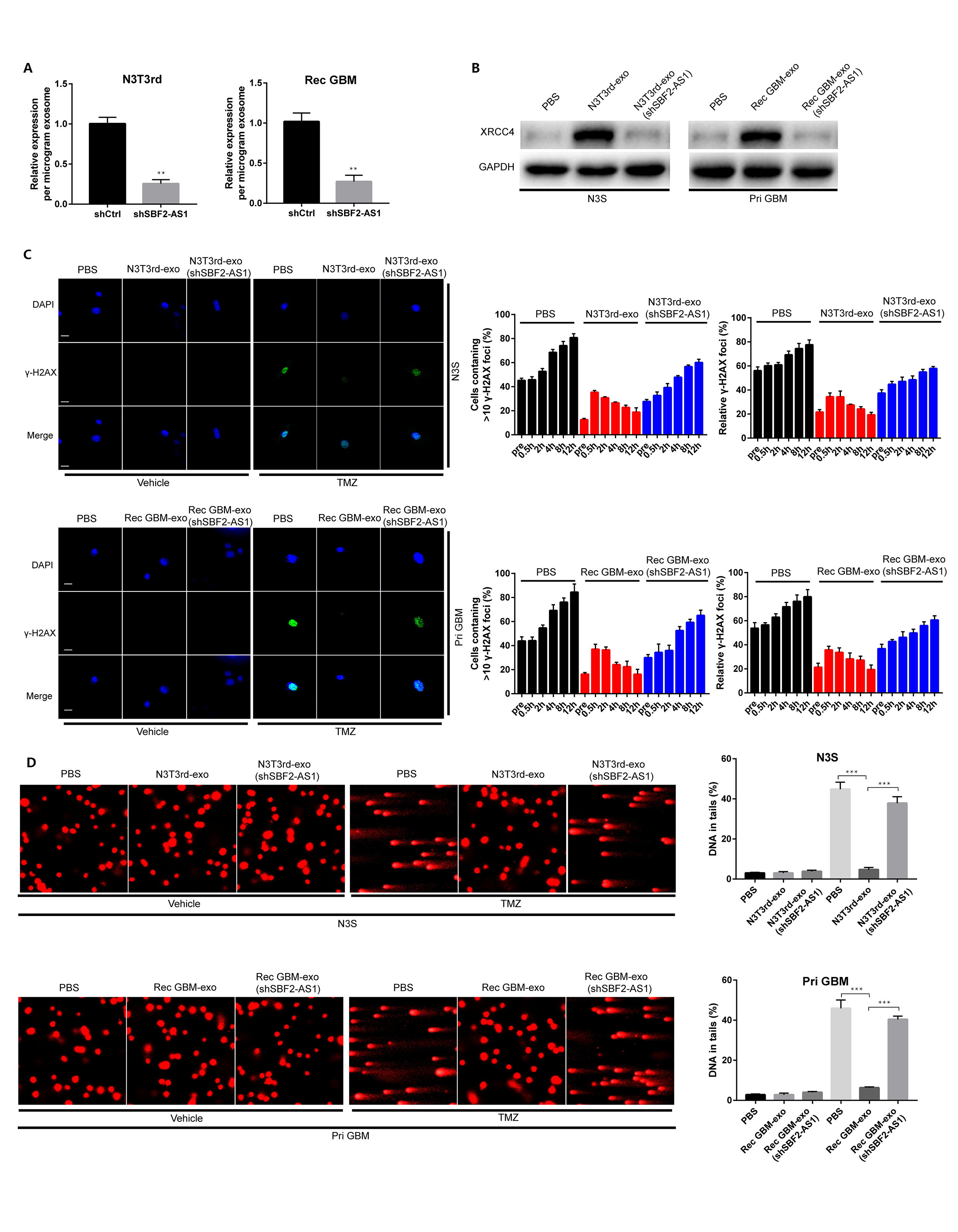

Supplement: Supplementary file 5 — Figure S4. A qRT-PCR analysis of lncSBF2-AS1 expression level in exosomes isolated from N3T3rd and Rec GBM cells, which were transfected shCtrl or shSBF2-AS1. The data represent the mean±SEM from three independent expriments. **P < 0.01 B Western blot assay for XRCC4 in Pri GBM and N3S cells treated with PBS, Rec GBM-exo/N3T3rd-exo or Rec GBM-exo (shSBF2-AS1)/N3T3rd-exo (shSBF2-AS1). GAPDH was used as the loading control. C Immunofluorescence staining of γ-H2AX foci in Rec GBM or N3T3rd cells which incubation with indicated exosomes for 2-day at 12h after TMZ exposure (200μM). Scale bar, 10μm. D Comet assay of Pri GBM and N3S cells treated with indicated exosomes at the indicated time after TMZ withdrawal. Data are means of three independent experiments±SEM. **P < 0.01. Scale bar, 50μm. (TIF 1722 kb) [file 13046_2019_1139_MOESM5_ESM.tif]
