# Supplementary material for: Exosomal transfer of long non-coding RNA SBF2-AS1 enhances chemoresistance to temozolomide in glioblastoma
Source: J Exp Clin Cancer Res. 2019 Apr 16;38:166. doi: 10.1186/s13046-019-1139-6 (PMC6469146; doi:10.1186/s13046-019-1139-6)
Supplement: Supplementary file 6 — Table S1. Twenty GBM patients and treatment characteristic. Table S2. Primers for qRT-PCR and siRNA target squence. Table S3. Clinicopathological features of 20 GBM patients and treatment characteristic. Table S4. Clinicopathological features of GBM patients in TCGA database. (DOCX 24 kb) [file 13046_2019_1139_MOESM6_ESM.docx]

**Supplementary table 1. Twenty GBM patients and treatment characteristic**

| **Characteristic** | **Value** |
| --- | --- |
| **Total samples (n)** | 20 |
| **Sex (n)** |  |
| Male | 11 |
| Female | 9 |
| **Medium age, years (range)** | 57 (22-74) |
| **Tumor location** |  |
| Frontal | 13 |
| Non-frontal | 7 |
| **Medium KPS (range)** | 80 (30-90) |
| **MGMT promotor status** |  |
| Methylated | 9 |
| Unmethylated | 11 |
| **IDH1/2 genotype** |  |
| Mutation | 6 |
| Wild-type | 14 |
| **Recurrent time (days)** |  |
| ≥300 | 9 |
| ＜300 | 11 |
| GBM1 (figure. 1E) | 335 |
| GBM2 (figure. 1E) | 447 |
| **TMZ dose** | 75 mg/m^2^/d after first surgery |

Abbreviations: KPS, Karnofsky performance status; MGMT, O-6-methylguanine-DNA-methyltransferase; IDH1/2, isocitrate dehydrogenase 1 and 2; TMZ, temozolomide.

**Supplementary table S2. Primers for qRT-PCR and siRNA target squence.**

| **Primers used for quantitative RT-PCR** |  |  |
| --- | --- | --- |
| **Gene** | **Forward-primer (5’-3’)** | **Reverse-primer****(5’-3’)** |
| SBF2-AS1 | CCACGACCCAGAAGGAGTCT | GCATTGATGGAGCATTGCGA |
| ZEB1 | AATCAGCCAGATCCCTCCCT | ATTGAGGGGCGAGGGAAAAG |
| XRCC4 | GGCCTGATTCTTCACTACCTGA | AAAGAGGTCTTCTGGGCTGC |
| miR-151a-3p | ATAAGATCTGGCTGCAACCCGTGTTC | ATAGTCGACCTCAGTGGAGCATTCC |
| Loc101593348 | ATTACAACCTCCGCGTGGTC | CCATCTGGGTCTGTGAAACCA |
| RP11-111F5.4 | CGAACTGCCACTGTCCTGAT | GGGGTCTTGGGCTGAGTAAA |
| LINC00271 | GGTGTCTGAGGTGGTGTAGC | CACCACACAGGCACTGCTTA |
| PSMD5-AS1 | GCTTCATGGTTAAGGGGGCT | GGCAGGCCTACTGACTCAAG |
| XLOC-003734 | TCCCCAGCCATACGGAACTA | AGATGTGGTCTTTGGCTCCC |
| RP11-348P10.2 | CTCGCCTGAGTGTCTCATCC | TCGATGGGTGTCCAATGTCG |
| CTC-480C2.1 | CTGCGCAATGCGTTATGGTC | GCAAACTCAGTCTCGGTCCC |
| ARHGEF26-AS1 | CCTCATGGAGCCGTGAGTTC | GCCTGATCACGCTGATGTCT |
| U6 | CTCGCTTCGGCAGCACA | AACGCTTCACGAATTTGCGT |
| β-actin | GTCATTCCAAATATGAGATGCGT | GCATTACATAATTTACACGAAAGCA |

| **Primers used for CHIP experiments** | | | |
| --- | --- | --- | --- |
| **Gene** | **Binding site** | **Forward-primer (5’-3’)** | **Reverse-primer (5’-3’)** |
| **SBF2-AS1** | **Site 1** | CCAATCAGGCCTTTAGGGCA | GACTCTGAAGTCCAGGCTCC |
|  | **Site 2** | ACTTAATGGGCCTGTCGCTG | AGCCTTGAATGGCTGACCAA |
|  | **Site 3** | ACCGCTGATGAACTTTCCCA | TGGAACTACCACAACGAGGC |
|  | **Site 4** | CGTCCCCTAAGGCCAATACC | AGCGTATGCATTTCGTTGCC |

| **Sequence for siRNA and shRNA** | |
| --- | --- |
| **Name** |  |
| **SBF2-AS1 shRNA-1** | GGGCATAAGCAGGATGGATTG |
| **SBF2-AS1 shRNA-2** | GCCAGTTTGCTGACAGCAACC |
| **SBF2-AS1 shRNA-3** | GCTATCACCAGGCCTGAATTC |
| **ZEB1 siRNA** | GGAAGAGGAGGAGGATAAA |
| **XRCC4 siRNA** | GCATGGACTGGGACAGTTT |

**Supplementary table 3. Clinicopathological features of 20 GBM patients and treatment characteristic**

| **Characteristic** | **All patient** | |
| --- | --- | --- |
|  | Low  lncSBF2-AS1  (n=10) | High  lncSBF2-AS1  (n=10) |
| **Type** |  |  |
| Pri GBM | 8 | 3 |
| Rec GBM | 2 | 7 |
| **Sex (n)** |  | |
| Male | 6 | 7 |
| Female | 4 | 3 |
| **age** |  |  |
| ≥45 | 8 | 7 |
| ＜45 | 2 | 3 |
| **Tumor location** |  | |
| Frontal | 4 | 6 |
| Non-frontal | 6 | 4 |
| **KPS score** |  |  |
| ≥80 | 7 | 6 |
| ＜80 | 3 | 4 |
| **MGMT promotor status** |  | |
| Methylated | 4 | 8 |
| Unmethylated | 6 | 2 |
| **Extent of surgery** |  |  |
| Total | 6 | 7 |
| Subtotal | 4 | 3 |
| **IDH1/2 genotype** |  | |
| Mutation | 1 | 2 |
| Wild-type | 9 | 8 |
| **ERBT does (Gy)** | 60 | |
| **TMZ dose** | 75 mg/m^2^/d after first surgery | |
| **Cut-off value (Median)** | 1.856208503 | |

Abbreviations: KPS, Karnofsky performance status; MGMT, O-6-methylguanine-DNA-methyltransferase; IDH1/2, isocitrate dehydrogenase 1 and 2; TMZ, temozolomide.

**Supplementary table 4. Clinicopathological features of GBM patients in TCGA database**

| **Characteristic** | **All patient** | |  |
| --- | --- | --- | --- |
|  | Low  lncSBF2-AS1  (n=77) | High  lncSBF2-AS1  (n=77) |  |
| **Sex (n)** |  |  |  |
| Male | 47 | 53 |  |
| Female | 30 | 24 |  |
| **age** |  | |  |
| ≥45 | 64 | 72 |  |
| ＜45 | 13 | 3 |  |
| **Cancer status** |  |  |  |
| With tumor | 65 | 61 |  |
| Tumor free | 12 | 16 |  |
| **KPS score** |  | |  |
| ≥80 | 43 | 39 |  |
| ＜80 | 34 | 38 |  |
| **Radiation therapy** |  |  |  |
| Yes | 68 | 65 |  |
| No | 9 | 12 |  |
| **TMZ chemotherapy** |  | |  |
| Yes | 50 | 54 |  |
| No | 27 | 23 |  |
| **Subtype** |  |  |  |
| Classical | 19 | 20 |  |
| Mesenchymal | 33 | 23 |  |
| Neural | 7 | 19 |  |
| Proneural | 25 | 15 |  |
| **Recurrent** | 3 | 10 |  |
| **Cut-off value (Median)** | 0.353979366 | |  |

Abbreviations: KPS, Karnofsky performance status; TMZ, temozolomide.
